# Dual-site catalysts featuring platinum-group-metal atoms on copper shapes boost hydrocarbon formations in electrocatalytic CO_2_ reduction

**DOI:** 10.1038/s41467-023-38777-y

**Published:** 2023-05-27

**Authors:** Manjeet Chhetri, Mingyu Wan, Zehua Jin, John Yeager, Case Sandor, Conner Rapp, Hui Wang, Sungsik Lee, Cameron J. Bodenschatz, Michael J. Zachman, Fanglin Che, Ming Yang

**Affiliations:** 1 Department of Chemical and Biomolecular Engineering, Clemson University, Clemson, SC USA; 2 Department of Chemical Engineering, University of Massachusetts Lowell, Lowell, MA USA; 3 Institute for New Energy Materials and Low Carbon Technology, Tianjin University of Technology, Tianjin, China; 4 X-ray Science Division, Argonne National Laboratory, Lemont, IL USA; 5 Environmental Effects and Coatings Branch, NASA John H. Glenn Research Center, Cleveland, OH USA; 6 Center for Nanophase Materials Sciences, Oak Ridge National Laboratory, Oak Ridge, TN USA

**Keywords:** Chemical engineering, Nanoparticles, Electrocatalysis, Materials for energy and catalysis

## Abstract

Copper-based catalyst is uniquely positioned to catalyze the hydrocarbon formations through electrochemical CO_2_ reduction. The catalyst design freedom is limited for alloying copper with H-affinitive elements represented by platinum group metals because the latter would easily drive the hydrogen evolution reaction to override CO_2_ reduction. We report an adept design of anchoring atomically dispersed platinum group metal species on both polycrystalline and shape-controlled Cu catalysts, which now promote targeted CO_2_ reduction reaction while frustrating the undesired hydrogen evolution reaction. Notably, alloys with similar metal formulations but comprising small platinum or palladium clusters would fail this objective. With an appreciable amount of CO-Pd_1_ moieties on copper surfaces, facile CO^*^ hydrogenation to CHO^*^ or CO-CHO^*^ coupling is now viable as one of the main pathways on Cu(111) or Cu(100) to selectively produce CH_4_ or C_2_H_4_ through Pd-Cu dual-site pathways. The work broadens copper alloying choices for CO_2_ reduction in aqueous phases.

## Abstract (web summary)

The inclusion of platinum-group metals for CO2 reduction electrocatalyst design may trigger the unwanted hydrogen evolution reaction. However, here the authors show that single-atom Pd and Pt on facet-selective Cu can selectively boost CO2 to CH4 or C2H4 conversion through dual-site pathways.

## Introduction

Among the catalytic reactions that can hydrogenate CO_2_ into energy-intensive hydrocarbon molecules (HCs), the electrocatalytic CO_2_ reduction reaction (CO_2_RR) featuring CO_2_ hydrogenation without producing H_2_ gas in any prior steps is of particular interest. While there are ample choices of elements to catalyze CO_2_RR into CO and formate molecules^1^, copper (Cu) is the demonstrated element that can effectively catalyze the HCs formation (especially C_2+_) owing to its optimal binding energies for both hydrogen (ΔE_H_^*^) and carbonyl species (ΔE_CO_^*^)^2,3^, the ubiquitous intermediate during CO_2_RR. Copper, therefore, serves as a unique catalytic metal to not only enable the circular carbon economy on earth but to extend human life beyond earth’s orbit through in situ utilization of the in-space CO_2_ to make methane as fuel or ethylene as building block molecules.

Metal alloying is a broadly employed strategy to accelerate the CO_2_RR while reasonably suppressing the competitive hydrogen evolution reaction (HER)^4,5^. Metals such as Ag, Au, Pb, and Sn that are either in the same group or to the right of Cu in the periodic table have been extensively studied^6^. These metals, with a typical positive H^*^ binding energy (>0.5 eV), are the well-accepted desired alloying choices with copper due to their unfavorable energetics toward the HER^3^. However, limitations are inevitable on the product side - these alloyed metals naturally render weakened ΔE_CO_^*^ and thus limit the main products to either formate or CO molecules^7–10^, defeating the initial purpose of using Cu as a catalytic metal to enable deeper hydrogenation of CO_2_.

If HER is never a concern, the above limitations can be straightforwardly addressed by incorporating platinum group metals (PGMs) residing on the left side of Cu in the periodic table, as the PGMs would provide substantial binding energy for CO^*^. However, many studies suggest that incorporating PGM components, such as nanoparticles and clusters, into Cu catalysts often results in penalties for enhanced HER along with improved conversion rates of carbon-based products, if any^11–15^. While it is indisputable that populating the catalyst surface with CO^*^ reduces the amount of H^*^ and the chance for subsequent H-H coupling to produce hydrogen gas^16–20^, HER is still, kinetically, a considerably faster reaction on many PGM moieties as compared to CO_2_RR (2e^-^ transfer vs. multi-electron transfer reaction)^21^. This pitfall was addressed in a series of recent works^22–24^ at a device level by incorporating catalysts on PTFE-based gas diffusion electrode (GDE) or catalyst-PFSA heterojunction arrangement as the electrode (by decoupling the gas-ion-electron transport process) to mitigate the HER tendency. In this work, from the perspective of designing the PGM-Cu catalytic center itself, we set out to find an alternative structure of the PGM-Cu interface that can favor CO_2_ hydrogenation to HCs through Cu but intrinsically limit HER. To unfold this possibility, we propose that two critical aspects of nanocatalyst design shall be satisfied simultaneously: (1) the PGM species anchored on the Cu host must be structured differently as opposed to extended PGM surfaces known to promote HER; (2) the PGM species as identified in aspect#1, must utilize the nearby Cu surfaces to render the enrichment of CO^*^ for further hydrogenation.

Herein, we report the design, synthesis, and characterizations of Pd_1_Cu (as the primary research target) and Pt_1_Cu single-atom alloys (SAAs) nanocatalysts for facile CH_4_ and C_2_H_4_ formation through CO_2_RR. With a trace amount of PGMs acting as the CO^*^ promoting sites and the preserved CO_2_ hydrogenation chemistry on Cu, promising results were made from the SAAs. Through shape-controlled catalyst synthesis, in-situ reaction studies, and DFT calculations, we demonstrate how PGM_1_Cu SAAs, as dual-site catalysts, enhance CO_2_RR performance without disrupting the HC formation capacity created by their parent Cu catalysts.

## Results

### Design of PGM_1_-Cu SAAs materials

To begin, we performed the work based on polycrystalline Cu nanoparticles (NPs) to reflect the general relevance of the work and then we developed specific shape-controlled catalysts for reaction mechanistic-oriented studies. We used galvanic displacement (GD) to synthesize SAAs using M^2+^ salts (M = Pd^2+^, Pt^2+^) to replace the elemental Cu, referred to as Pd_1_Cu SAA and Pt_1_Cu SAA. We adopted a modified wet chemical method^25,26^ using ascorbic acid as a reducing agent and antioxidant for Cu NPs, instead of using commercial Cu directly. This approach minimizes the presence of Cu_2_O on the parent Cu surface, as found in commercial Cu (Supplementary Fig. S1a and associated discussion), thus circumventing the potential negative effects of Cu_2_O on the synthesis of SAA and the resulting catalytic activity. By accurately controlling the concentration of the PGM salts and the time of exchange during the GD synthesis (to be discussed soon), we made a series of SAA catalysts favoring the HCs yields over the HER side reaction during the CO_2_RR. In this work, we mainly discuss the characterizations and CO_2_RR activity of Pd_1_Cu and highlight about Pt_1_Cu when appropriate for discussion.

**Fig. 1:**
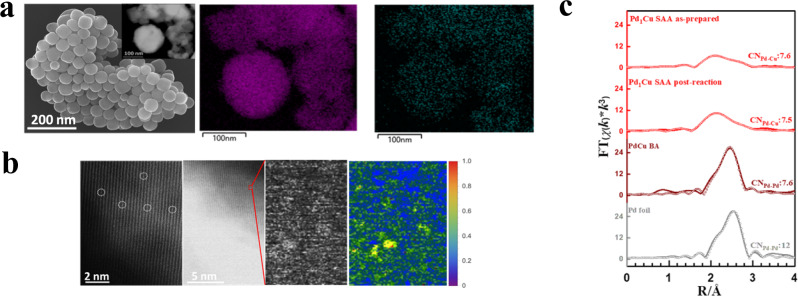
Morphology and structure analyses of polycrystalline PGM-Cu SAAs. **a** FESEM image of the polycrystalline Cu catalyst and the inset shows the Pd_1_Cu SAA along with its corresponding EDS elemental map (right) showing the atomic distribution of Pd on the parent Cu surface. **b** Aberration-corrected HAADF-STEM images for the Pd_1_Cu SAA (left). The circles highlight the single-atom Pd. Enlarged and colorized intensity map of the highlighted red square region showing isolated Pd atoms (center): yellow-red regions in the color contour represent Pd atomic dispersion and blue represents Cu surface (right). **c** Pd K-edge EXAFS (shadowed lines) and the curve-fit (points) for Pd_1_Cu SAA as-prepared, Pd_1_Cu SAA post-reaction, PdCu BA, and Pd foil is shown in *R*-space.

To begin the investigation, field emission scanning electron microscopy (FESEM) and high-resolution transmission electron microscopy (HR-TEM, insert) in Fig. 1a shows a polycrystalline nature of a generic Cu-based catalyst ranging from 50–150 nm (Supplementary Fig. S1b). Next, energy dispersive spectroscopy (EDS) elemental mapping, as shown in Fig. 1a, indicates the presence of Pd in a highly dispersed state, and its dilute concentration with the Cu host is 0.9 wt.% according to ICP analyses (Supplementary Table S1). Further, the Pd moieties were identified as single-atom species on the lattice of Cu by aberration-corrected high angle annular dark-field scanning transmission electron microscopy (HAADF-STEM) (Fig. 1b and Supplementary Fig. S2). We analyzed the Pd K-edge of the Pd_1_Cu SAA as-prepared/post-reaction catalysts by the extended X-ray absorption fine structure (EXAFS). As evident from Fig. 1c and Supplementary Table S2, the Pd atoms in these Pd_1_Cu SAA samples are stabilized through the Pd-Cu coordination in the first shell at a radial distance of ~2.60 Å. The possible contribution of Pd-Pd coordination near 2.74 Å is minimal in these SAA as-prepared and post-reaction samples, indicating the Pd atoms are isolated by the surrounding Cu atoms to avoid aggregations even after the CO_2_RR. These first-shell Pd-Cu and Pd-Pd distances agree well with the previous studies^27–29^. In addition, the consistent Pd-Cu coordination around 7.5 (CN = 12 is usually for bulk alloys) in both as-prepared and post-reaction SAA catalysts suggests that single-atom Pd species reside on the catalyst surface and the local coordination environment for the Pd experiences minimal change after the reaction, indicating the promising stability of the SAA structure.

To compare with the SAAs made by the GD method, we also synthesized dilute PdCu bimetallic alloys (BAs) through the conventional coprecipitation approach by using a dilute amount of Pd precursor and NaBH_4_ as a reducing agent. The NaBH_4_-induced reduction leads to preferred Pd-Pd bond formation with BA structure resembling nanoparticulate or cluster forms. In contrast to the single-atom Pd nature in the Pd_1_Cu SAA catalyst, we observe a CN_Pd-Pd_ of 7.6 for the PdCu BA, suggesting the presence of small Pd nanoclusters^30^ in the BA catalyst as our reference sample designs intended (Fig. 1c and Supplementary Table S2). The ICP data (Supplementary Table S1) and CO_2_RR reactivity screening results (Supplementary Fig. S3a) reveal that, even with similar or lower concentrations of Pd, the incorporated Pd in the BA catalysts fails to deliver any significantly improved HCs formation or suppressed HER. We observed similar behaviors upon characterizing the Pt_1_Cu system (Supplementary Tables S1 and Figs. S3b & S4). These results indicate that a general formulation of dilute PGMs in Cu is not a guarantee for the improved catalytic performance to be discussed shortly in this report.

### Enhanced CO_2_RR to HCs and Reduced HER by PGM_1_-Cu SAAs

**Fig. 2:**
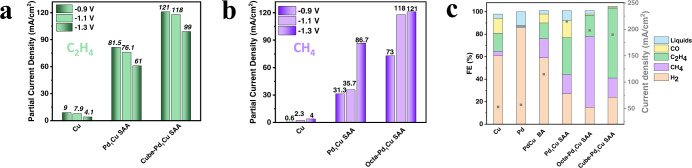
Electrocatalytic CO_2_ reduction activity comparison. The partial current density of **a** C_2_H_4_ and **b** CH_4_ at different voltages (*vs*. RHE) for polycrystalline Cu, polycrystalline Pd_1_Cu SAA, and shape-controlled Pd_1_Cu SAAs. **c** Comparison of CO_2_ reduction FE% and current densities of Pd_1_Cu SAAs and their key counterparts. FE% analysis was performed by running an amperometric *i-t* curve at −1.1 V *vs*. RHE and quantifying the products over 30 min. The catalyst loading on the GDE was maintained at about 220 µg/cm^2^ for these tests.

We tested the CO_2_RR activity in a flow-cell electrolyzer (3-compartment cell with 1cm^2^ gas diffusion electrode (Supplementary Figs. S5 and S6) using CO_2_-saturated 0.5 M KHCO_3_ as electrolyte and CO_2_ gas feed as the reactant). All the reported potentials are referenced to the reversible hydrogen electrode (RHE) unless specified otherwise. Among the key samples, there were negligible liquid products and a similar amount of gaseous CO formation (Supplementary Figs. S7 and S8 and Table S3). To begin with the conversion rate, at −0.9 V, the polycrystalline Pd_1_Cu SAA exhibits multi-fold improved CH_4_ and C_2_H_4_ partial current densities as compared to its parent polycrystalline Cu NPs (Fig. 2a, b). This improvement is dramatic in part because the catalyst loading on the GDE was maintained at about 220 µg/cm^2^. When a much higher (1 mg/ cm^2^) catalyst loading was used, the reactivity difference between the samples shrank to 2–3 times due to the more significant mass transfer limitations to the SAA samples. On the reaction selectivity, as compared to Cu NPs, the Pd_1_Cu SAA shows significant improvement in HCs products (Supplementary Fig. S7 for itemized columns at −1.1 V). The Faradaic efficiency (FE) of CH_4_ increases from 6 to 25% and the C_2_H_4_ from 16 to 33% from the Cu NPs to Pd_1_Cu SAA. Notably, with an almost identical chemical formulation, the bimetallic alloy PdCu BA fails to deliver any similar improvement for CH_4_ and C_2_H_4_ production in terms of FE and current densities when measured against the Pd_1_Cu SAA. Worse, the creation of Pd-Pd, even in small clusters, in the PdCu BA sample leads to a doubled FE for HER. As expected, the HER becomes the single-dominant reaction in the case of Pd-only nanoparticles. By estimating the electrochemically active surface area (ECSA) that is proportional to the double-layer capacitance of the catalysts (Supplementary Fig. S9), we confirmed that the galvanic exchange preparation method does not improve the ECSA to give rise to the reactivity boost.

We have further made shape-controlled Cube Pd_1_Cu SAA and Octahedral (Octa- in short) Pd_1_Cu SAA to improve the admittedly mediocre performance of the initial polycrystalline catalysts in terms of partial current density and FE for CH_4_ or C_2_H_4_ formation (Fig. 2 and Supplementary Fig. S3), and more importantly, to bridge to mechanistic studies in later sections of this report. Since Cu(100) faceted (Cube surface) and Cu(111) faceted (Octahedra surface) crystals yield C_2_H_4_ and CH_4_ as major products^31^, respectively, we leveraged the Pd_1_Cu SAA recipe to enhance the CO_2_RR performance towards either CH_4_ or C_2_H_4_ selectively. These shape-specific SAAs are stable with a partial current density of ca. 100 mA/cm^2^ and FE > 50% for a reaction duration of over 300 min (Supplementary Fig. S10).

We observed a similar encouraging trend when adopting Pt besides Pd through the SAA strategy for reaction tests using 0.5 M KHCO_3_ (Supplementary Figs. S3b, S8, S11). These Pd_1_- and Pt_1_-Cu SAAs rank among the highly promising PGM-containing catalysts for CO_2_RR in making HCs (Supplementary Table S4 and Fig. S12a, b). Although the commercial Cu (particle size 40–60 nm, Sigma Aldrich) seems to be a better platform than our home-made Cu to achieve appreciable CH_4_ and C_2_H_4_ formations, we were unable to use the commercial Cu as the starting material to produce the SAA catalyst, as discussed earlier (Supplementary Fig. S1a). Nevertheless, we benchmarked the Pd_1_Cu SAA using the inferior homemade Cu with the advanced commercial Cu sample, and we are still able to confirm the enhanced activity from the SAA (Supplementary Fig. S13a). Besides the alloy catalytic chemistry, the surface pH increase at higher current densities may well be the external contributor toward the limited HER and enhanced HCs formation. Hence, we performed additional reaction studies for the Cu and Pd_1_-Cu SAA catalysts with different electrolyte pHs (and estimated surface pHs) at the same ionic strength. While the higher pH indeed helps the reactions of interest, the Pd_1_-Cu SAA catalysis is clearly the more significant contributor (Supplementary Fig. S13b and associated discussion).

We caution readers that the present work intends to improve the CO_2_RR performance from the aspect of nanocatalyst engineering for mediocre metallic copper catalysts made in-house, so the reported activity boost shall not be regarded as the ultimate high performance for any Cu-based catalysts, especially at the device level. More detailed discussion on this matter can be found in the *Discussion* section. Additionally, the dramatic differences in the catalytic performances, as we observed, are not a result of the overall catalyst particle size changes, as indicated by the similar size of Cu NPs with or without displaced PGM shown in Fig. 1a and Supplementary Figs. S1a, S2, and S4. It has been previously reported that the difference in particle size influences the CO_2_RR product selectivity^32,33^. However, in our experiment, the influence of particle sizes with narrower distribution on reaction selectivity won’t be explicitly reflected. The above results show that to steer CO_2_RR towards more HCs without compromises for favoring HER simultaneously, it is beneficial to include PGM atoms in the catalysts but most likely only in the form of isolated PGM atoms.

### Desired CO^*^ and H^*^ Interactions of PGM_1_Cu SAA

**Fig. 3:**
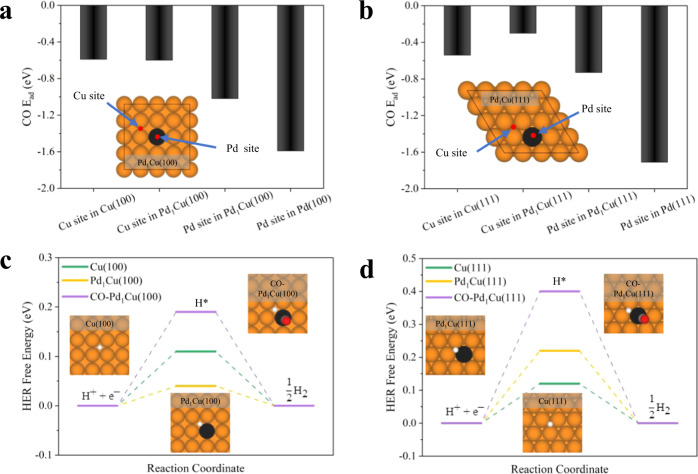
DFT calculations on the role of single-atom Pd in Pd_1_Cu SAA on tuning the CO^*^ adsorption and HER. **a**, **b** show the adsorption energy of CO^*^ over (100) and (111) facets of bare Cu, Pd_1_Cu, and bare Pd, respectively. The top views of Pd_1_Cu(100) and (111) with their possible CO^*^ adsorption sites (red) are inserted. **c**, **d** are the free energy diagram of HER over (100) and (111) facets of bare Cu, Pd_1_Cu, and Pd_1_Cu with the presence of a CO molecule adsorption over the Pd site (CO-Pd_1_Cu). The HER free energy was calculated under standard conditions. The configurations of H^*^ on these three scenarios are inserted. Color code: Cu (orange), Pd (black), C (gray), O (red) and H (white).

We performed DFT calculations and examined the role of Pd_1_Cu SAA interfacing with the ubiquitous CO_2_RR early intermediates, namely CO^*^ and H^*^ on catalyst surfaces^3,34,35^ (for details of DFT calculations, see Methods, Supplementary Information, and Fig. S14). Pd_1_Cu(100) and Pd_1_Cu(111) surfaces were selected for DFT calculations. This is because the computational supercells of Pd_1_Cu(111) and Pd_1_Cu(100) matched reasonably well with the experimental shape-controlled samples in terms of averaged Pd-Cu distance (2.61 Å for Pd_1_Cu(111), 2.66 Å for Pd_1_Cu(100) in theory vs. ~2.61 Å for Octa Pd_1_Cu SAA and ~2.65 Å on average for Cube-Pd_1_Cu SAA in experimental. see Supplementary Table S2) and Pd-Cu coordination number (9 for Pd_1_Cu(111), 8 for Pd_1_Cu(100) in theory vs. ~8.7 for Octa Pd_1_Cu SAA and ~7.6 on average for Cube-Pd_1_Cu SAA in experimental, see Supplementary Table S2). For CO^*^ over the Pd_1_Cu(100) surface (Fig. 3a), we studied different adsorption sites (Supplementary Table S5), including Cu site, Pd site and Pd-Cu interfacial sites. We found that CO^*^ over the Pd_top site has the most favorable adsorption energy of −1.02 eV, which is stronger than that on Cu(100) of −0.59 eV but weaker than that on Pd(100) of −1.59 eV. The adsorption energy values indicate that the presence of the single-atom Pd could improve CO^*^ adsorption significantly compared to the bare Cu(100). It is worth mentioning that the most favorable CO^*^ adsorption energy on the Cu site of Pd_1_Cu(100) is nearly the same as that of Cu(100). This means the single-atom Pd doesn’t influence the CO^*^ adsorption performance over the nearby Cu atoms. We obtained similar results for the Pd_1_Cu(111) surface (Fig. 3b). Among all sites of Pd_1_Cu(111) (Fig. 3 and Supplementary Table S6), CO^*^ over the Pd-top site has the strongest CO^*^ adsorption energy of −0.73 eV, which is larger than that on Cu(111) of −0.54 eV but weaker than that on Pd(111) of −1.71 eV. When CO^*^ adsorbs over the Cu site of Pd_1_Cu(111), its adsorption energy is slightly weaker than the pure Cu(111) surface by ~0.2 eV. Thus, for a typical Pd_1_Cu SAA, the Cu site behaves like pure Cu for CO^*^ adsorption, and the Pd_1_ site renders stable adsorption of CO^*^.

To probe the translational effect, we also performed the CO^*^ adsorption on the Pt_1_Cu SAA surfaces (Supplementary Fig. S15 and Tables S5–S6). The DFT results show that the Pt_1_ site significantly strengthens the CO^*^ adsorption, similar to the Pd_1_ site. The most favorable adsorption sites of CO^*^ are the Pt_top sites, with adsorption energies of −1.42 eV and −1.06 eV over Pt_1_Cu(100) and Pt_1_Cu(111) surfaces, respectively. The results suggest that the single-atom PGM (i.e., Pd, Pt) on the Cu matrix plays a similar role in strengthening the CO^*^ adsorption and potentially achieving a higher CO^*^ surface coverage than the pure Cu surfaces. To quantify the impact of the single-atom PGM on the binding and the surface coverage of CO, we calculated the adsorption free energy of adding CO one by one on Pd_1_Cu(100) and Cu(100) surfaces (Supplementary Fig. S16). Our results indicate that when CO adsorbed over the PGM of Cu-based SAA, the adsorption free energy of CO is significantly higher (~0.4 eV) than that of the pure Cu, and the PGM didn’t change the adsorption free energies of other CO adsorbates over the Cu sites of SAAs. Thus, SAAs can potentially increase the CO surface coverage and the degree of the increment of the CO surface coverage depends on the densities of the PGM of SAAs. The overly strong binding of carbonyl species may inhibit the subsequent hydrogenation steps. Considering the dilute atomic dispersion of PGM on Cu surface, notorious CO poisoning is expected not to be an issue in our case. Toward this objective, we performed CO stripping experiments for Pt_1_Cu SAA, PtCu BA, Cu-only, and Pt-only catalysts (Supplementary Fig. S17). We chose Pt over Pd for this study to correlate better the hydrogen underpotential deposition (H_UPD_) and CO adsorption properties on the catalyst surface due to the distinguishable signals of Pt as compared to that of Pd for CO adsorption/desorption. The results suggest that the Pt_1_Cu SAA catalyst, behaving almost like the Cu-only reference in this experiment, has remarkable anti-CO-poisoning capability when measured against the Pt-only and PtCu BA counterparts.

How does the PGM_1_Cu SAA hold for HER activity? We investigated the role of the Pd site in Pd_1_Cu by examining the free energy of HER. To construct the free energy diagram of HER, we considered two scenarios, including H^*^ over the clean Pd_1_Cu SAA (Supplementary Tables S7 and S8) and H^*^ over the Pd_1_Cu SAA with one pre-existing CO^*^ on the top site of Pd (Fig. 3c, d). The results show that when there is a pre-existing CO^*^ on the top site of Pd of the Pd_1_Cu SAA, the free energies of HER over Pd_1_Cu SAA are increased by 0.08 eV and 0.28 eV compared to Cu(100) and Cu(111) and thus, HER is suppressed. This finding is also translational to Pt_1_Cu SAA (Supplementary Tables S7–S8). The free energy diagram for HER (Supplementary Fig. S15c, d) shows that when there is a pre-existing CO^*^ on the top site of Pt of the Pt_1_Cu SAA, the free energies of HER over the Pt_1_Cu(100) and Pt_1_Cu(111) SAA increase by 0.20 eV and 0.42 eV compared to those over Cu(100) and Cu(111), respectively. Therefore, DFT results suggest that HER could be suppressed thermodynamically by PGM_1_Cu SAA due to the lateral interaction raised by strong, co-adsorbed CO^*^ over the PGM top site. This is consistent with the previous theoretical studies that an improved CO^*^ coverage and a stronger CO^*^ adsorption energy could weaken H^*^ adsorption and reduce HER^14–18^. Our theoretical results are also in agreement with our experimental findings that Pd_1_Cu significantly suppresses the FE of HER as compared to the pure Cu catalyst under CO_2_RR conditions. Indeed, in the event of non-CO_2_RR reaction condition, as the DFT results suggested, the PGM_1_Cu configurations may promote the HER compared with the parent Cu-only surfaces, and our blank reaction test using Ar instead of CO_2_ in the gaseous feed stream confirmed this prediction as well (Supplementary Figs. S7 and S8).

### Mechanistic Interrogation of Pd_1_Cu SAAs for CO_2_RR

**Fig. 4:**
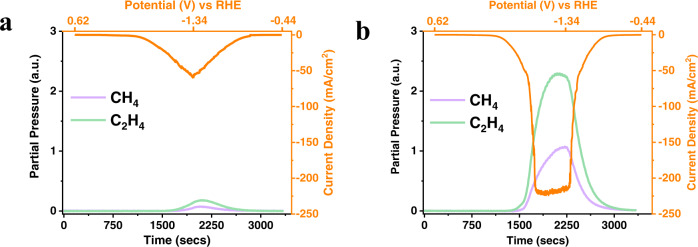
Real-time analysis of gaseous products as a function of scanning potential. Cyclic voltammograms and real-time hydrocarbon product distribution detected by mass spectrometer upon CO_2_ reduction using **a** parent Cu NPs and **b** Pd_1_Cu SAA. The potential was scanned at 1 mV/sec, and mass spectrometer data points were collected every 4 s providing the matching time interval of the online analysis of products as a function of voltage. The measurement was started after reaching the steady-state condition. Potential is scanned from open-circuit voltage to −1.34 V (vs. RHE) and then reversed back to −0.44 V (vs. RHE). (a.u.: arbitrary units).

Figure 4 shows the evolution of products by slow voltage scanning from open-circuit voltage to −1.34 V and then back to −0.44 V through our online CV-Mass spectrometer setup. Compared with the parent Cu-only catalyst, we observe an earlier onset of CH_4_ and C_2_H_4_ by scanning the potential in a more negative direction for the Pd_1_Cu SAA catalyst, which drives the rapid increase in current density as the HCs formation becomes more significant. Similar phenomena can be observed on Pt_1_Cu SAA catalyst, as shown in Supplementary Fig. S18. These results indicate that the PGMs enrich the catalyst surface with crucial reaction intermediates leading to facile reactions under the CO_2_RR condition.

**Fig. 5:**
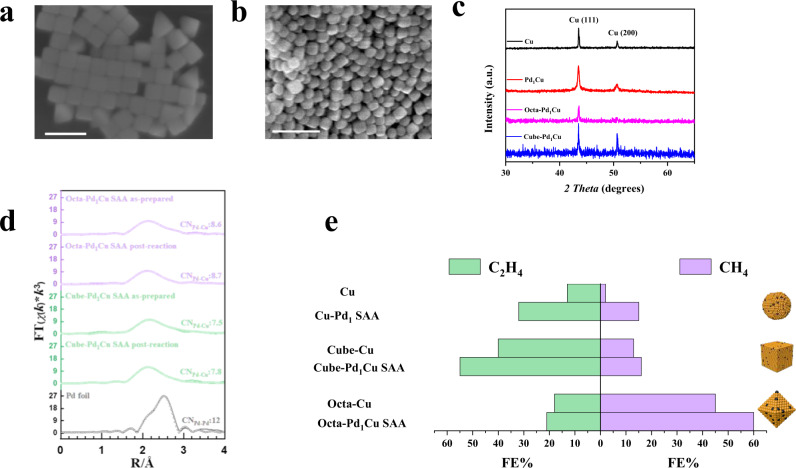
Effect of Pd on Cu for product distribution during CO_2_ reduction. SEM image of **a** Cube-Cu and **b** Octa-Cu. The scale bar is 200 nm. **c** Powder X-Ray diffraction patterns of polycrystalline and shape-controlled Cu nanoparticles and Pd_1_Cu SAAs. (a.u.: arbitrary units). **d** Pd K-edge EXAFS (shadowed lines) and the curve-fit (points) for Octa-Pd_1_Cu SAA as-prepared, Octa-Pd_1_Cu SAA post-reaction, Cube-Pd_1_Cu SAA as-prepared, Cube-Pd_1_Cu SAA post-reaction, and Pd foil are shown in *R*-space. The data are k^3^-weighted and not phase-corrected. **e** The FE distribution of CH_4_ and C_2_H_4_ obtained using Cu and Pd_1_Cu SAAs (at −1.1 V *vs*. RHE).

We benchmarked these findings by synthesizing Cu cubes exposing Cu(100) facet and Cu octahedra exposing Cu(111) facet (Fig. 5a–c) and alloying reaction-stable atomically dispersed Pd species on these Cu shapes (Fig. 5d for EXAFS, Supplementary Fig. S19 for elemental mapping). Since Cu(100) faceted (Cube surface) and Cu(111) faceted (octahedra surface) crystals yield C_2_H_4_ and CH_4_ as major products, respectively^31^, we leveraged our Pd_1_Cu SAA recipe to enhance the CO_2_RR performance of these shape-controlled catalysts toward either CH_4_ or C_2_H_4_ selectively (Supplementary Fig. S20). As discussed earlier, these shape-controlled Pd_1_Cu SAAs are advantageous to the polycrystalline SAAs (Fig. 2c), and they rank among the highly promising PGM-containing catalysts for CO_2_RR in making HCs (Supplementary Table S4 and S12a and S12b). With the help of the single-atom Pd, these shape-controlled Pd_1_Cu SAAs also show enhanced FE and improved partial current density for targeted products than their parent Cu-only cube and octahedra samples (Supplementary Fig. S20). Perhaps the more exciting fundamental observation is that the Cube-Pd_1_Cu SAA and Octa-Pd_1_Cu SAA specifically enhances the CH_4_ and C_2_H_4_ formation, respectively, without altering the intrinsic selectivity of their parent Cube-Cu and Octa-Cu samples (Fig. 5e). The observation of preserved Cu catalytic property after doping is unusual yet desired in CO_2_RR research for HC formations, where the alloying strategy with various types of the second metal often leads to overhauls of reactivity and selectivity^7–10^.

To probe the impact of the SAA on reaction pathways for CO_2_RR by copper, we conducted the in-situ ATR-SEIRAS at −0.8 and −1.2 V for polycrystalline Cu NPs and Pd_1_Cu SAA as the initial comparisons. Supplementary Fig. S21 and Table S9 show the time evolution of the absorbance peaks mainly between 1000 to 1800 cm^−1^, where the peak assignments were based on the collective information from recent literature^36–38^. While the polycrystalline Cu NPs lagged the polycrystalline Pd_1_Cu SAA in terms of absorbance peak evolutions as a function of applied voltage and reaction time, both samples exhibit similar patterns in terms of wavenumbers in the IR spectra, suggesting the reaction pathway for the catalysts could be similar. Speaking of the promotion effect, SAA helps in decreasing the overpotential for the formation of a variety of intermediates. At −0.8 V, the onset of CO_2_ reduction is observed over polycrystalline SAA as indicated by the evolution of peaks for COOH^*^, which is one of the early intermediates for CO_2_RR toward CO_2_ hydrogenation, but such species cannot be clearly detected until −1.2 V over Cu NPs. Similarly, the formations of CHO^*^, OCCHO^*^, OCCO^*^, and OCH_2_^*^, indicative of C_2_H_4_ and CH_4_ formations and albeit appearing as a mixed package in the spectra, have already proceeded over SAA at −0.8 V, whereas −1.2 V would be required for the polycrystalline Cu NPs.

**Fig. 6:**
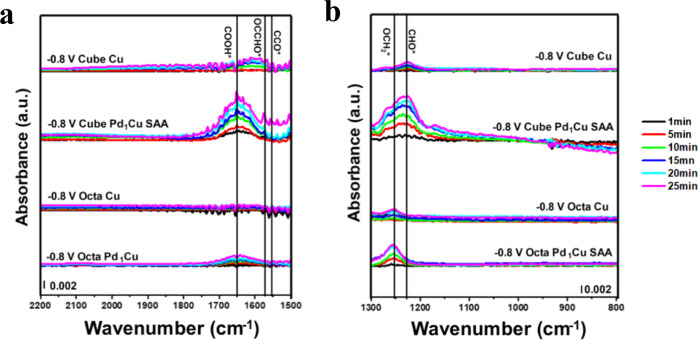
in-situ ATR-SEIRAS of shape-controlled Cu and Pd_1_Cu SAA. The spectra **a** at higher wavenumbers and **b** at lower wavenumbers collected as a function of time for shape-controlled Pd_1_Cu SAA and shape-controlled Cu in CO_2_-saturated 0.5 M KHCO_3_ electrolyte at −0.8 V. Spectra situated between 1300 and 1500 cm^−1^ were omitted because of data distortions and overlaps caused by (bi)carbonate species. For easier readability and format consistency, we put the *toward the end of the reaction intermediates in this report, and readers shall defer to the computational models to determine the actual terminal atom for those adsorbates. To ensure that the study of the reaction mechanism by identification of intermediates using ATR-SEIRAS was not compromised by structural and morphological transformations of catalyst, we performed TEM and XRD analyses of cube-based samples as-prepared/post-reaction. The results summarized in Fig. S22 suggest that overall the catalysts are stable under the period of our ATR-SEIRAS test. (a.u.: arbitrary units).

A similar trend in the ATR-SEIRAS can be tracked in the shape-controlled Cu and SAA catalysts as shown in Fig. 6, indicating that the introduced single-atom Pd on shape-controlled Cu will not necessarily create completely new reaction pathways but promote the CO_2_RR reaction based on the parent copper. Importantly, the ATR-SEIRAS studies on these shape-controlled samples allow us to gain better resolution to distinguish the otherwise mixed CH_4_ and C_2_H_4_ formation routes displayed by the polycrystalline samples. As the reaction test result suggested (Fig. 5e and Supplementary Fig. S20), Octa-Cu and Octa-Pd_1_Cu SAA catalysts favorably catalyze the CH_4_ formation in CO_2_RR. In the corresponding ATR-SEIRAS spectra of these two Octa-series samples, an OCH_2_^*^ characteristic peak can be detected around 1250 cm^−1^, and this is one of the main intermediates for CH_4_ production. In the meantime, it is hard to detect C-C coupling intermediates such as OCCHO^*^ at 1580 cm^−1^ and OCCO^*^ at 1560 cm^−1^ in these catalysts. Differently, Cube Cu and Cube Pd_1_Cu SAA catalyze the C_2_H_4_ formation favorably (Fig. 5e and Supplementary Fig. S20). In the corresponding ATR-SEIRAS spectra of these Cube-series samples, OCCHO^*^ (more prominent) and OCCO^*^ intermediates from C-C coupling can be detected at 1580 and 1560 cm^−1^, respectively. As the precursor intermediates to these C-C coupling intermediates, CHO^*^ at 1230 cm^−1^ and COH^*^ at 1160 cm^−1^ can also be detected. In comparison, OCCHO^*^ and its precursor CHO^*^ have more noticeable abundancy compared to OCCO^*^ and COH^*^, indicating OCCHO^*^ is a more prevalent C-C coupling in our catalysts and testing conditions. As an additional minor point, the Cube-series samples display a shoulder peak at slightly higher wavenumbers than the designated OCH_2_^*^ for CH_4_ formation in the Octa-series samples, and we cautiously attribute this minor shoulder peak to OCH_2_^*^-related C_2_ oxygenate radicals toward ethylene formation^39^.

Our DFT results agree with the above experimental observations. When CO^*^ hydrogenates through a surface H^*^ and the produced intermediate CHO^*^ is thermodynamically more favorable than COH^*^ over all examined surfaces (i.e., Cu(100), Cu(111), Pd_1_Cu(100) and Pd_1_Cu(111)) (Supplementary Fig. S23 and Tables S10–S13). The reasons that we followed the Langmuir-Hinshelwood (LH) mechanism (CO^*^ hydrogenates through a surface H^*^) is that (1) Our ATR-SEIRAS results (Fig. 6, Supplementary Fig. S21, and Table S9) suggest a clearly more prominent CHO^*^ rather than COH^*^ as the C_1_ intermediate over the Cu-based SAA surfaces when using KHCO_3_ solution as the electrolyte and a previous theoretical study showed that CO hydrogenation tends to form CHO^*^ by the surface H^*^^40^, (2) The LH mechanism is also consistent with the kinetic order study of the electrochemical CORR-to-CH_4_^41^ i.e., the CO hydrogenation had a negative reaction order since CO^*^ and H^*^ are in competition with each other for surface sites and this does not agree with the Eley-Rideal (ER) mechanism, in which a positive reaction order in the partial pressure of CO should be expected.

We acknowledge that the intermediates involved in C-C coupling are still under debate. Some studies consider CO dimerization^36,37^, while some other studies contemplate CO-CHO coupling^42,43^. In this work, we followed CO-CHO coupling because we observed more abundant CHO^*^ and OCCHO^*^ species from the in-situ ATR-SEIRAS experiment for the Cube-series catalysts that favor ethylene formation. In addition, we performed DFT calculations to further confirm this C-C coupling process (i.e., CO dimerization, CO-CHO coupling) by comparing the reaction energy of both CO^*^ + CHO^*^ → OCCHO^*^ + * and CO^*^ + CO^*^ → OCCO^*^ + * (Supplementary Fig. S24). Our results show that CO-CHO coupling is more thermodynamically favorable than CO dimerization.

**Fig. 7:**
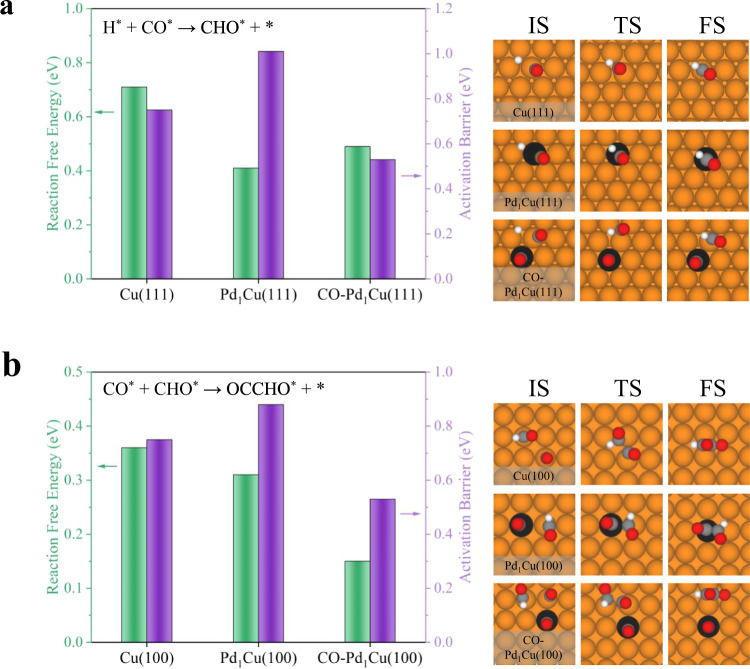
DFT calculations on the role of single-atom Pd in Pd_1_Cu SAA on reaction free energy and activation barrier. **a** The reaction free energies and activation barriers of CO^*^ hydrogenation (H^*^ + CO^*^ → CHO^*^ + *) on Cu(111), Pd_1_Cu(111) and CO-Pd_1_Cu(111) with the corresponding structures of IS, TS and FS. **b** The reaction free energies and activation barriers of C-C coupling (CO^*^ + CHO^*^ → OCCHO^*^ + *) on Cu(100), Pd_1_Cu(100) and CO-Pd_1_Cu(100) with the corresponding structures of IS, TS and FS. All the free energy and activation barrier were calculated at room temperature.

When the reactions are “forced” to occur on the single-atom Pd site of the clean Pd_1_Cu SAA surfaces, the reaction free energies of CO^*^ hydrogenation and C-C coupling decreases, but their activation barriers increase, compared to those steps over pure Cu (Fig. 7 and Supplementary Table S14). This indicates that although the single-atom Pd site within the SAA surface could favor the thermodynamic energies of the potential RDSs of CO_2_RR to hydrocarbons, their kinetic properties could be inhibited since the corresponding activation barriers significantly increased. However, as a consequence of the Pd sites in Pd_1_Cu SAA populating the surface with CO^*^ (CO-Pd_1_Cu(111)), both the reaction free energy and activation barriers of CO^*^ hydrogenation to CHO^*^ are 0.21 eV and 0.22 eV lower over the Cu sites of the CO-Pd_1_Cu(111) catalyst compared to those over the Cu(111), respectively (Fig. 7a). We obtained similar results of C-C coupling over CO-Pd_1_Cu(100) (Fig. 7b) which suggests that the CO^*^ adsorption over the Pd site of SAA induces the surface lateral interaction and destabilizes the adsorption of the initial state (IS) of CO^*^ hydrogenation and C-C coupling steps more than that of the transition state (TS) and final state (FS). Similar to the Pd_1_Cu SAA, with a pre-existing CO^*^ on the top of the Pt site of Pt_1_Cu SAA, the reaction free energies of CO^*^ hydrogenation and C-C coupling over the Cu site of CO-Pt_1_Cu SAA also become more thermodynamically favorable compared to those over the bare Cu catalysts (Supplementary Figs. S15 and S25). Taken together, this work paves the way for loading of atomically dispersed PGM atoms on single-crystal Cu surfaces in future endeavors, and we conclude that the promotion effect of single-atom PGM (i.e., Pd, Pt) on the Cu matrix on the CO_2_RR can be promising.

## Discussion

We adopted a wet chemical approach for synthesizing polycrystalline Cu NPs with a wide particle size distribution (50–150 nm range). The CO_2_RR activity exhibited by our polycrystalline Cu NPs is thus lower than some of the engineered Cu materials tested in flow-cell configurations. These reported high performances were achieved by engineering copper catalysts to introduce grain boundaries as the new active sites^44,45^ or optimizing the catalyst layer at a device level through vacuum deposition techniques and catalyst-ionomer heterojunctions^22,46–48^. As the overall reaction performance is sensitive to various factors beyond catalytic materials design, we acknowledge these above advancements, and we reiterate that this work focuses on tailoring PGM chemistry on metallic copper nanocatalysts (see XPS results in Supplementary Fig. S26). We caution readers that the multi-fold increase in partial current density for HCs reported for the polycrystalline Pd_1_Cu SAA catalyst at −1.1 V *vs*. RHE using 0.5 M KHCO_3_ electrolyte shall not be considered as absolute optimal performances, because tuning parameters such as catalyst loading, cell configuration, voltages, and electrolyte will further induce profound influences on the reaction performances. In addition, there are intertwined effects even during the reaction, where the more active SAA catalysts will indeed bring up the local pH and the higher local pH will further favor the targeted reaction. We were able to decouple these two impacts by showing that the alloying effect is major and the pH effect is minor (Supplementary Fig. S13b and associated discussion). Still, readers shall be cautious about how to interpret individual reactivity data, and the alloy vs. pH effects shall not be generalized.

In line with a recent review by ref. ^6^, the secondary metals that show weaker H binding than Cu gain natural popularity in the established dilute Cu alloy research. We draw a corollary between our work and other exciting efforts of developing Cu-based alloys to display the merits. For example, Sn is generally believed to induce weaker H binding and stronger O binding than Cu in CO_2_RR. As a consequence of varying Cu:Sn bulk compositions, the Sn-abundant surfaces have been shown to selectively produce formate, while the single-atom Sn on Cu favors CO formation^10,49^. In another example, using single-atom Pb to alloy with Cu, Zheng et al. found that the Pb dopant regulated the Cu to preferentially adsorb HCOO^*^ over COOH^*^ during the CO_2_RR and thus favored formate formation^9^. Perhaps due to the “co-introduction” synthesis approaches, a good portion of these metals were introduced to the bulk of the Cu. These secondary metal atoms throughout the surface and the bulk of the Cu host modified the catalytic property of Cu, and the obtained catalysts made products other than HCs. To that end, the goal of designing active and selective alloy catalysts for making deeply hydrogenated HCs products remains elusive^50^. The Pd_1_Cu and Pt_1_Cu SAAs developed in this work, unexpectedly, would not agitate the HER reaction, but significantly promote the selective HCs formation in connection with respective facet control of the Cu hosts. On the better understood thermal catalytic system, the original concept of single-atom alloy catalysis was launched by Sykes and Stephanopoulos groups almost a decade ago^51–53^, where the PGM_1_Cu allows facile activation of incoming H_2_ on the PGM site and enables the selective hydrogenation on Cu surfaces. Now, the weak or largely absent proof^54^ of the exact single-atom alloy concept in electrocatalytic hydrogenation has been identified and extended (e.g., HER mitigation and tunable host metal effects).

We also acknowledge the recent reports showing that perhaps the most effective catalytic centers for CO_2_RR by Cu catalysts reside on the interfaces of different facets, edges, or corner sites of truncated nanoparticles^55,56^. By anchoring PGMs exclusively at these specific locations instead of the entire surfaces as we did in this study, one may achieve intriguing reaction performances by using even less amount of PGMs in the interest of cost reduction. However, the present experimental and theoretical work designs were not intended for speculated site-specific PGM-Cu systems. Understanding precisely how the site-specific PGM-Cu chemistry will behave in the alternative but the related platform is worth a detailed study underway. The synthesis procedure adopted by us has the possibility of introducing other metal dopants and modifications of structures (both atomically and morphologically surface restructuring). While these factors are crucial, our studies on shape-controlled Cu-only and SAA catalysts suggest the overall reliability of our results in focusing on the PGM-Cu chemistry. A strong indication is that our shape-selective nanocatalysts displaying predominantly (111) and (100) facets delivered reaction selectivity agreeing well with the previously obtained selectivity results with cubes and octahedra for CO_2_RR in literature^31,57,58^.

Although it is out of the scope of the present work, we believe systematic single crystal studies originating from vacuum to model reactions (with operando pump-probe IR or Raman) would offer the best chance to avoid data noises (due to defects and impurities) and to interrogate the detailed reaction kinetics, mechanism, and the possible influence of atomic structure rearrangement caused by the galvanic exchange between Pd and Cu. In the meantime, well-defined Cu surfaces and crystals may undergo surface structural changes during the CO_2_RR reaction within a relatively brief time (e.g., a few hours)^59,60^. Such a risk factor must be carefully considered and mitigated for detailed surface science studies. In the future, to gain a deeper understanding of the single-atom PGM effect on CO_2_RR, we aim to develop a multi-scale, multi-physics simulation that quantitatively considers the electrode potential, pH, intermediate, and alloy effects.

We show in this work that platinum group metals, albeit being traditionally unfavored for electrocatalytic CO_2_ hydrogenation, can now be leveraged as alloyed single atoms on the Cu matrix to dramatically improve the reaction efficiency to selectively produce hydrocarbon molecules, which is a reaction target that many other Cu-based alloys won’t be able to achieve. With the PGM-heavy automotive catalysts phasing out under the megatrend of vehicle electrification, this newly found application of PGM catalysis unfolds unique opportunities for the global PGM market and workforce.

## Methods

### Cu Nanoparticles

We prepared polycrystalline Copper nanoparticles by wet chemical synthesis method using ascorbic acid (AA) and ethylene glycol (EG). AA acts as both a reducing agent as well an antioxidant. Thus, during the synthesis process, it avoids the formation of Cu oxides. AA can scavenge the free radicals and the dissolved oxygen in the reaction mixture giving it dual functionality. Typically, 319 mg of CuSO_4_ (2 mmol) was dissolved in 100 mL ethylene glycol under vigorous stirring in the N_2_ atmosphere. We maintained the temperature of the solution at 80˚C. 275 mg (2.5 mmol) of Polyvinylpyrrolidone (PVP-40K) was added to this and continued to stir until a clear solution. Separately, we dissolved 2.5 mmol of PVP and 2.8 g (16 mmol) of Ascorbic acid in 100 mL of EG until a clear solution. We prepared both solutions under a nitrogen atmosphere and vigorous stirring at 80˚C. The two solutions were mixed and allowed to react for 30 mins. 2 mmol of NaBH_4_ was added to the final dispersion to reduce the unreacted Cu^2+^ to form 50–150 nm nanoparticles. The solution was collected in 50 mL tubes and centrifuged at 11,627 × *g* for 20 min. The Cu nanoparticles were washed with deionized water and ethanol three times each to obtain PVP-free Cu nanoparticles.

### Cu shapes

We used the solvothermal method to synthesize the facet selective Cu nanoparticles.

#### Cubes

21 mL of oleyl amine (OLAM) was degassed under vacuum until bubbling of dissolved gases stopped. We increased the temperature to 80 ˚C and switched the gas flow to N_2_ to maintain the inert atmosphere. 430 mg (3 mmol) of CuBr was added and vigorously stirred until dissolved under N_2_ flow. We added 1.16 g (3 mmol) of trioctylphosphine oxide (TOPO) and allowed it to dissolve. The temperature was then increased to 260˚C, keeping the inert atmosphere intact. The reaction was allowed for 2 h at 260 ˚C to form 50–120 nm nanoparticles, after which we cooled it with compressed gas. The resulting solution was quickly transferred to tubes and centrifuged at 11,627 × *g* for 20 min. We washed the product with hexane three times and re-dispersed it in O_2_-free hexane for storage.

#### Octahedra

48 mL of OLAM was degassed under vacuum until a gas bubble stopped appearing to remove dissolved gasses. We switched the Schlenk line to N_2_ gas flow and increased the temperature to 80 ˚C. After 2 h, we increased the temperature to 335 ˚C. Separately and simultaneously, 395 mg of CuCl (4 mmol) and 1.78 mL (4 mmol) of Tri-octyl phosphine (TOP) were heated to 200 ˚C under N_2_ for 2 h to form the TOP-Cu complex. This complex was quickly and carefully added to hot OLAM and allowed to react for 30 min to form 40–200 nm nanoparticles. We cooled the mixture under compressed air flow and promptly collected the product in tubes. They were centrifuged at 11,627 × *g* for 20 min and then washed three times with Toluene. Finally, we stored the Cu octahedra in O_2_-free toluene for future use.

### Galvanic displacement for SAA preparations

We performed rigorous initial optimizations by varying concentration, time, and reaction conditions to obtain homogeneously dispersed PGM metals on Cu Surface.

#### Pd_1_Cu SAA

We prepared 50 mM H_2_PdCl_4_ by dissolving the 1:2 ratio of PdCl_2_ and Conc. HCl at 60 °C. For the galvanic displacement, 30 mg of Cu nanoparticle was dispersed in 10 mL DI water under N_2_ protection. We added 60 µL of the stock H_2_PdCl_4_ (50 mM) to the dispersion and stirred the mixture for 30 min under N_2_ atmosphere. The final concentration of Pd used was 0.3 mM in the dispersion. After the reaction, the dispersion was centrifuged at 10k rpm for 20 min and washed with ethanol. We collected the first supernatant liquid for elemental analysis. The resulting powder after centrifugation is the Pd_1_Cu SAA. We used the same procedure to obtain SAA of facet selective Cu morphology (cubes and octahedra).

#### Pt_1_Cu SAA

We dispersed 30 mg of Cu nanoparticle in 10 mL of DI water under N_2_ atmosphere. An aqueous solution of tetraamine Platinum (II) Nitrate [Pt(NH_3_)_4_(NO_3_)_2_] was added to this so that the total concentration of Pt^2+^ is 0.3 mM. We stirred the dispersion for 30 min under N_2_ flow. The product was collected by centrifugation and washing with ethanol. We also collected the supernatant for elemental analysis.

#### PGMCu BA (M= Pd, Pt) bimetallic cluster alloys

We synthesized these sets of samples by wet chemical reduction instead of galvanic displacement reaction. 75 mg (0.47 mmol) of CuSO_4_ was dissolved in 10 mL DI water, and we added aqueous solutions of H_2_PdCl_4_ or Pt(NH_3_)_4_(NO_3_)_2_ to keep the total concentration of Pd^2+^ or Pt^2+^ at 0.3 mM under stirring in the N_2_ atmosphere. Then, we added 75 mg (2 mmol) of NaBH_4_ to reduce the metal ions and form nanoparticles. The product was collected by centrifugation using the procedure mentioned above. For varied M (Pt and Pd): Cu ratios, we used different amounts of metal salts in the process. Among the various BA catalysts (Supplementary Table S1), the bimetallic alloy closely resembling the concentration of PGM in the SAA catalyst was used to compare the CO_2_ reduction activity in the main text.

### Morphology and composition study

The elemental composition of obtained samples was analyzed using ICP-OES (Thermo Fisher iCAP 7200 ICP-OES Analyzer) and Energy Dispersion X-ray Spectroscopy (EDS). XRD patterns were collected using an X-ray diffractometer (Rigaku D/max 2500) at a scan rate of 1° min^–1^ in the 2θ range of 10–70°. We recorded the Field emission scanning electron microscopy (FE-SEM) images using a Hitachi SU9000 CFE SEM/STEM with a resolution of 0.4 nm at 30 kV (SE). We obtained TEM images and SAED patterns using the Hitachi HT7830 UHR 120 kV instrument with a resolution of 0.14 nm. We recorded Atomic-resolution aberration-corrected HAADF-STEM images and EDS mappings using JEOL JEM-ARM2100F equipped with a CEOS probe corrector. Preliminary We collected XAS data at Beamline 12-BM, Argonne National Laboratory (APS); Pd K-edge absorption spectra were acquired using a water-cooled double Si (311) monochromator. We recorded the spectra in the fluorescence mode with a 7-element silicon drift detector (SDD, Vortex ME-7, Hitachi). For the EXAFS analysis of samples after electrochemical reaction, catalyst coated GDL were treated under typical CO_2_RR reaction conditions (0.5 M KHCO_3_ as an electrolyte and at –1.1 V (vs. RHE)) for 30 min. BF-STEM images of the post-reaction sample were acquired in the Center for Nanophase Materials Sciences (CNMS) at Oak Ridge National Laboratory (ORNL) on a Nion UltraSTEM 100. The instrument was operated at 60 kV with a semiconvergence angle of ~31 mrad.

### Electrochemical measurements

We performed all electrocatalytic CO_2_ reduction, and in-situ ATR-SEIRAS experiments on a CHI 760E workstation. For the initial investigations, a custom-built 60 mL two-compartment H-cell was used with a Sustanion membrane (Dioxide Materials) as the separating membrane between the counter and working electrode compartment. We fabricated the working electrode by drop-drying 200 µL of catalyst dispersion (30 mg in 3 mL ethanol) on a pre-wet (with ethanol) gas diffusion electrode (Sigracet 39BB) with loading at ca. 200–250 µg/cm^2^. We studied the CO_2_RR activity of the samples in a continuous flow cell reactor with Pt (1 cm^2^) foil as counter and Ag/AgCl (sat. KCl) as reference electrode. We maintained the total feed gas flow was at 23 sccm and the electrolyte circulation flow rate was 4 mL/min. The flow rate reported here is the gas flow rate at the cell outlet. The flow rates from the reactor outlet are almost identical (< ± 5%, within the error of MFCs) to the inlet flow rates set by the mass flow meters. Therefore, we did not apply flow rate corrections when using KHCO_3_ solution as the electrolyte^61,62^. However, we caution that this approximation cannot be universally applied, where the accumulative impact of CO_2_ loss, reaction stoichiometry governed volume change, and overall conversion could become significant even when the inlet and outlet gas flow temperatures are the same. Thus, the flow rate correction becomes a must in such a case. Flow rate corrections became necessary for alternative tests where the KOH electrolyte has been used as CO_2_ loss was significant. The geometric dimension of the active area of the working electrode is 1 cm^2^. Before electrochemical measurements, the whole reactor line was purged with Ar for 30 min and then with CO_2_ for 30 min to saturate the system with CO_2_. The open-circuit voltage was allowed to stabilize under these conditions. The outlet of the gas purging line was directed to a gas trap glassware and subsequently to an online mass spectrometer for gas product quantifications. The liquid products were quantified by collecting the electrolyte aliquots at different time intervals from catholyte flow and analyzed by ^1^D ^1^H NMR applying the water gate suppression technique (using DMSO as the internal standard). We calculated the Faradaic efficiency using the Eq. (1) as follows:

1 $${{{{{\rm{Faradaic}}}}}}\; {{{{{\rm{Efficiency}}}}}}\left(\%\right)=\frac{{{{{{\rm{moles}}}}}}\; {{{{{\rm{of}}}}}}\; {{{{{\rm{product}}}}}}\,\times n\times F}{I\times t}$$

Where n is the number of electrons required to form product from CO_2_ reduction in a balanced chemical equation (for example, *n* = 2 for CO, H_2_, *n* = 8 for CH_4_, etc.). F is the Faraday constant (96,485 C). I and t are the current and time from electrochemical biasing of working electrodes at different voltages. Potential is a reference to reversible hydrogen electrode by converting Ag/AgCl using Nernst equation as Eq. (2):

2 $${{{{{\rm{E}}}}}}\left({{{{{\rm{RHE}}}}}}\right)=E\left({{{{{\rm{Ag}}}}}}/{{{{{\rm{AgCl}}}}}}\right)+{E}^{0}\left({{{{{\rm{Ag}}}}}}/{{{{{\rm{AgCl}}}}}}\right)+0.059*{{{{{\rm{pH}}}}}}$$

As a control experiment, we performed reaction tests with Ar-only gas feed stream, while keeping the rest of the setups the same. H_2_ was the only product that we had detected in an appreciable amount (FE% over 95%, Supplementary Figs. S7 and S8). This validates that the hydrocarbon products during the CO_2_RR originate from the gaseous CO_2_ feed stream. For the EXAFS analysis of samples after the electrochemical reaction, catalyst-coated GDL were treated under typical CO_2_RR reaction conditions (0.5 M KHCO_3_ as an electrolyte and at –1.1 V (vs. RHE)) for 30 min. The Faradaic efficiency reported here is within the error range of ±5%.

### CO Stripping experiment

The CO stripping was performed by potentiostatically adsorbing the CO monolayer on the catalyst’s surface. 0.1 M H_2_SO_4_ was used as electrolyte to simultaneously monitor hydrogen underpotential deposition (H_UPD_) and CO adsorption. We performed the experiments using the catalyst deposited on the gas diffusion electrode. The electrolyte was purged with Argon for 30 min, and the catalyst surface was cleaned electrochemically by cyclic voltammetry between 0 V to 1 V (vs RHE). The electrode was then biased at 0.1 V (RHE) while purging CO gas (5% CO, balanced Ar) for 5 min to allow the adsorption of CO on the catalyst surface. After the potentiostatic adsorption, the electrolyte was re-purged with Ar to remove any dissolved CO in the electrolyte. We recorded the CV between 0 V and 1.3 V to observe any CO stripping peak and HUPD peaks in subsequent cycles.

### in-situ ATR-SEIRAS

in-situ ATR-SEIRAS: We performed SEIRAS experiments in a custom-built J1 Jackfish SEC cell with a face-angled crystal (FAC) as the working electrode substrate. The setup is compatible with PIKE VeeMAX III variable angle ATR sampling as used in the Thermo Nicolet iS50 FTIR instrument. First, a thin Au film was formed on the surface of Si FAC to prepare working electrodes. Then a catalyst ink was prepared by dispersing 10 mg of catalyst in 1 mL isopropanol, 50 µL of 5 wt% Nafion solution and sonication for 30 mins. 20 µL of this ink was drop-casted on the Au thin film-coated Si FAC surface and allowed to dry overnight. The experiments were performed in 60 mL airtight single chamber glassware with dedicated ports for gas bubblers, counter and reference electrodes, and exhaust traps. We used 0.5 M KHCO_3_ as an electrolyte, and before experiments, it was purged with N_2_ to remove any dissolved oxygen. We collected the baseline at −0.45 V, by saturating the electrolyte with CO_2_. We used Pt coil and Ag/AgCl (Sat. KCl) as a counter and reference electrode. We averaged 32 scans with a resolution of 4 cm^−1^ for the final SEIRAS data collection. The data was collected by biasing the working electrode at a specific voltage and recording the SEIRAS signal every 5 min (for 30 min) to see the peaks’ evolution patterns as a time function. In the plotted data, spectra situated between 1300 and 1500 cm^−1^ were omitted because of the reported and confirmed absorbance overlaps and distortions caused by (bi)carbonate species^36^.

### Computational details

We performed all density functional theory (DFT) calculations using Vienna Ab Initio Simulation Package (VASP) code. We applied the revised Perdew-Burke-Ernzerhof (RPBE) functional, and the projector augmented wave (PAW) method to treat exchange-correlation interactions in a periodic boundary system. We used the climbing image nudged elastic band (CI-NEB) and dimer methods to search for the transition state. For geometries considerations, we considered the configurations optimized when the energy had converged to 10^−4^ eV, and the forces were smaller than 0.03 eV/Å. We described the detailed theoretical approaches and results in the Supplementary Information.

## Supplementary information

Supplementary Information

## Data Availability

All data generated or analyzed during this study are included in this published article and its supplementary information files.
