# Supplementary material for: Dual-site catalysts featuring platinum-group-metal atoms on copper shapes boost hydrocarbon formations in electrocatalytic CO2 reduction
Source: Nat Commun. 2023 May 27;14:3075. doi: 10.1038/s41467-023-38777-y (PMC10224932; doi:10.1038/s41467-023-38777-y)
Supplement: Supplementary file 1 — Supplementary Information [file 41467_2023_38777_MOESM1_ESM.pdf]

Supplementary Information

Dual-site catalysts featuring platinum-group-metal atoms on copper shapes boost hydrocarbon formations in electrocatalytic CO_2 reduction

Manjeet Chhetri[1]†, Mingyu Wan[2]†, Zehua Jin[1]†, John Yeager[1], Case Sandor[1], Conner Rapp[1], Hui Wang[3], Sungsik Lee[4], Cameron J. Bodenschatz[5], Michael J. Zachman[6], Fanglin Che[2*], Ming Yang[1*]

[1]Department of Chemical and Biomolecular Engineering, Clemson University, Clemson, SC, USA. [2]Department of Chemical Engineering, University of Massachusetts Lowell, Lowell, MA, USA. [3]Institute for New Energy Materials and Low Carbon Technology, Tianjin University of Technology, Tianjin, China. [4]X-ray Science Division, Argonne National Laboratory, Lemont, IL, USA. [5]Environmental Effects and Coatings Branch, NASA John H. Glenn Research Center, Cleveland, OH, USA. [6]Center for Nanophase Materials Sciences, Oak Ridge National Laboratory, Oak Ridge, TN, USA. †These authors contributed equally to this work. *e-mail: myang3@clemson.edu; Fanglin_Che@uml.edu.

Inventory of Supplementary Information:

Supplementary Figures S1-S26,

Supplementary Tables S1-S14,

Electrochemically Active Surface Area Calculation,

Computational Setups,

XPS analysis of surface- Cu surface with and without Pd SAA inclusion,

Comments on effects of other parameters on Faradaic efficiency.

Supplementary Figures and Tables

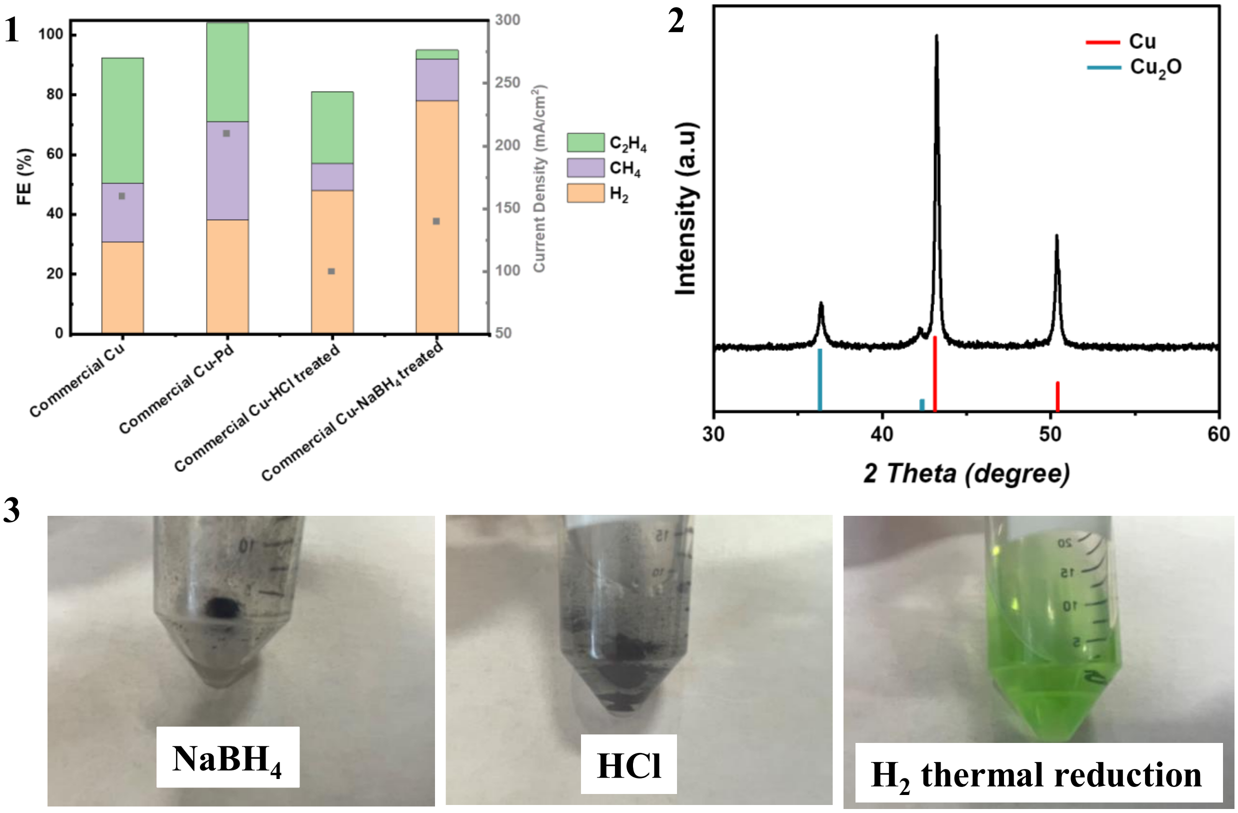

Supplementary Fig. S1a: Commercial Cu is not a good candidate as parent Cu for this study. 1 Comparison of CO_2RR FE% for various gas products and current densities obtained by using commercial Cu, Cu-Pd catalyst based on commercial Cu, and commercial Cu samples treated by HCl and $NaBH_4$ as catalysts. The catalyst loading on the GDE was maintained at 1 mg/cm^2, and the FE% and current density analyses were performed at -1.1 V *vs.* RHE in 0.5 M $KHCO_3$. **2** Powder X-Ray diffraction patterns of the commercial Cu sample. (a.u.: arbitrary units). **3** Optical images of final catalyst powders obtained by treating commercial Cu with $NaBH_4$ (2 mg $NaBH_4$ to 100 mg copper sample), HCl (pH~2), and H_2 thermal reduction (10 % H_2, 200 °C).

We tested the commercial Cu catalyst with a loading of 1 mg/cm^2, and we found good performance from this benchmark Cu as shown in Supplementary Fig. S1a. This would otherwise be a more capable Cu platform than the homemade Cu to develop our SAA catalysts. However, after introducing Pd onto this commercial copper, we observed a decrease in C_2H_4 FE to 33% and only about a 4% increment in C_2H_4 formation rate (partial current density for C_2H_4 production). In the meantime, increases in CH_4 and H_2 FE and partial current densities were observed. To understand this failed initial try, we found out that this was most likely caused by the presence of oxide phases in the commercial copper sample that was supposed to be metallic even with careful handling in the glove box. A substantial amount of copper(I) oxide was detected by XRD (Supplementary Fig. S1a-2) along with the metallic Cu. In our intended control synthesis, the Pd(II) cation will exchange with Cu(0) metallic solid to produce atomically dispersed Pd(0) as part of the alloy and the Cu(II) cation to leave. Differently, in this failed effort, when Pd(II) exchanges with Cu_2O, the copper host will stay as an oxide particle, leaving the reduced palladium to be either Pd(I) [1] embedded in the Cu_2O surface or unbound Pd(0) clusters (cannot stay as single-atom Pd due to high

surface energy). Therefore, we reason that during the galvanic exchange stage, the copper(I) oxide species may contribute to the formation of unwanted Pd structures under our current synthesis conditions.

We tried to remove the cuprous oxides in the commercial copper via either H_2 thermal reduction (10 % H_2, 200 ℃), $NaBH_4$ wet chemical reduction (2 mg $NaBH_4$ to 100 mg copper sample), and HCl dissolution (pH~2) to harvest metallic copper surfaces to allow the proper galvanic displacement of single-atom Pd(0) on Cu(0) surface akin to our initial experiments with Cu_2O free parent Cu nanoparticles. However, the collected powder products from those treatments did not look promising, as illustrated in Fig. SR1-1-1a-1 and a-2. Specifically, copper treated with $NaBH_4$ and HCl tended to aggregate in DI water, resulting in a significant decrease in activity; the copper reduced by H_2 was readily dissolved in DI water. Hence, we conclude that commercial copper is not a suitable platform for introducing single atoms in this study since it contains a substantial amount of Cu_2O species, and this species is hard to remove without altering other compositional and structural properties of the commercial copper sample.

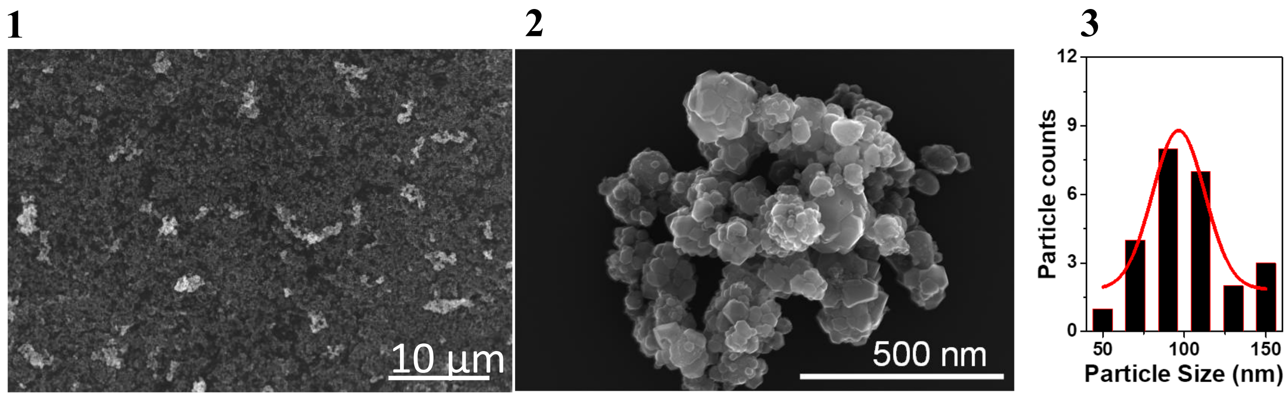

Supplementary Fig. S1b: Morphology analysis of Pd_1Cu SAA. 1 SEM image of the Pd_1Cu SAA catalyst on a gas diffusion electrode for reaction tests; **2** SEM image of the Pd_1Cu SAA catalyst and 3 Particle size distribution of polycrystalline Pd_1Cu SAA catalyst on the electrode substrate. The catalyst loading on the GDL is 200-220 µg/cm^2.

Supplementary Table S1: ICP-OES analysis of the screened samples

sample name	ICP-readings (ppm)			Formulation	wt.%
	Cu	Pd	Pt		
Pd_1Cu SAA	25.6	0.24	NA	$Cu_{25.6}Pd_{0.24}$	0.9
octa-Pd_1Cu SAA	145	0.57	NA	$Cu_{145}Pd_{0.57}$	0.4
Pt_1Cu-SAA	18.8	NA	0.22	$Cu_{18.8}Pt_{0.22}$	1.1
cube-Pd_1Cu SAA	271	0.98	NA	$Cu_{271}Pd_{0.98}$	0.36
CuPt-BA-6 mL	86.4	NA	0.86	$Cu_{86.4}Pt_{0.86}$	0.98
CuPt-BA-3 mL	78.5	NA	0.48	$Cu_{78.5}Pt_{0.48}$	0.6
CuPd-BA-0.48 mL	128.1	2.2	NA	$Cu_{128.1}Pd_{2.2}$	1.69
Cu-Pd-BA-0.24 mL	25.2	0.13	NA	$Cu_{25.2}Pd_{0.13}$	0.51
Cu-Pd-BA-5 mL	72.1	13.2	NA	$Cu_{72.1}Pd_{13.2}$	15.4

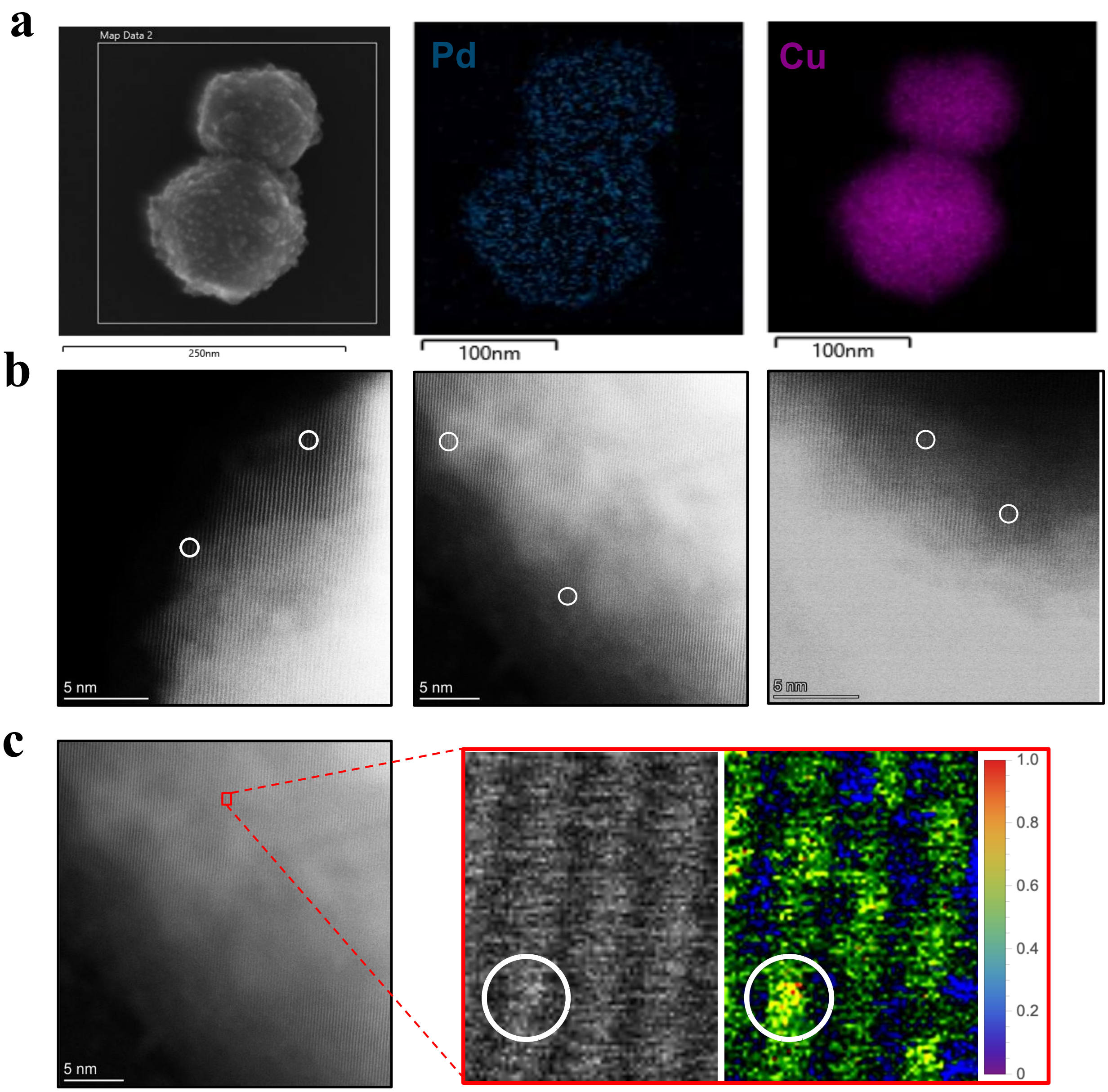

Supplementary Fig. S2: Morphology analysis of Pd_1Cu SAA. **a** STEM-EDS elemental mapping of the Pd_1Cu SAA catalyst, showing a uniform distribution of Cu and Pd elements. **b** HAADF-STEM images of the Pd_1Cu SAA catalyst. The white circle highlights the single-dispersed Pd atom. **c** HAADF-STEM images of the Pd_1Cu SAA catalyst, the red square of the highlighted region showing isolated Pd atoms

Supplementary Table S2: Curve-fit parameters for Pd K-edge EXAFS of catalysts. Pd foil, PdCu BA, Pd_1Cu SAA as prepared, Pd_1Cu SAA post-reaction, Octa-Pd_1Cu SAA as-prepared, Octa-Pd_1Cu SAA post-reaction, and Cube Pd_1Cu-SAA as-prepared, and Cube Pd_1Cu-SAA post-reaction.

Samples	Path	CN[a]	R(Å)[b]	R factor
Pd foil	Pd-Pd	12	2.74	0.5%
PdCu BA	Pd-Pd	7.6	2.58	0.1%
Pd_1Cu SAA as-prepared	Pd-Cu	7.6	2.58	0.1%
	Pd-Pd	0		
Pd_1Cu SAA post-reaction	Pd-Cu	7.5	2.61	0.9%
	Pd-Pd	0		
Octa-Pd_1Cu SAA as-prepared	Pd-Cu	8.6	2.60	0.0%
	Pd-Pd	0		
Octa-Pd_1Cu SAA post-reaction	Pd-Cu	8.7	2.61	0.2%
	Pd-Pd	0		
Cube-Pd_1Cu SAA as-prepared	Pd-Cu	7.5	2.64	0.7%
	Pd-Pd	0		
Cube-Pd_1Cu SAA post-reaction	Pd-Cu	7.8	2.67	0.0%
	Pd-Pd	0		

[a]Coordination number for the absorber-backscatterer pair. [b]Average absorber-backscatterer distance. 0.80 <s0<1, $\triangle E_0$ was refined as a global fit parameter, returning a value of no more than 10.0 eV. Data ranges: CN, ±20%; R, ±1%; 3.0 $\leq\Delta k\leq$12.9 Å, 1.0 $\leq\Delta R\leq$3.2 Å.

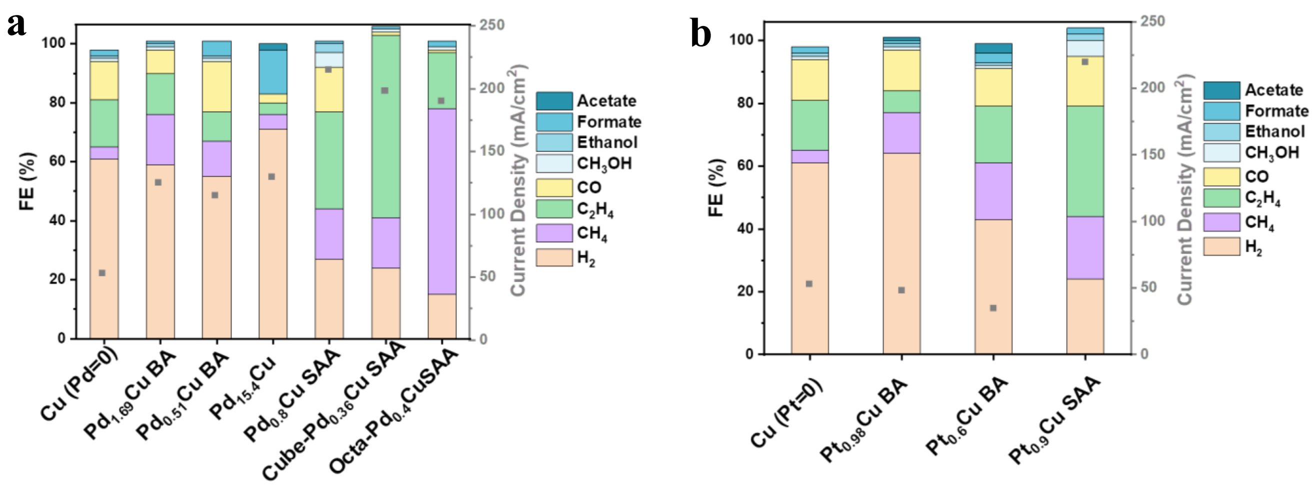

Supplementary Fig. S3: Comparison of M wt.% in Cu-catalyst with Cu-only, SAA, and BA samples for CO_2 reduction activity. a The FE comparisons of different products and current densities obtained by CO_2 electroreduction using different wt.% of Pd on Cu surface with different preparation methods. BA refers to the catalysts obtained by chemically reducing Cu^{2+} and M^{2+} (M=Pd/Pt) using $NaBH_4$ as a reducing agent during synthesis. SAA refers to catalysts obtained by reducing Cu^{2+} by galvanically displacing it with M (Pd/Pt). **b** The FE comparisons of different products and current densities obtained by CO_2 electroreduction using different wt.% of Pt on Cu surface with described above two methods. The catalyst loading on the GDE was maintained at 220 μg/cm^2.

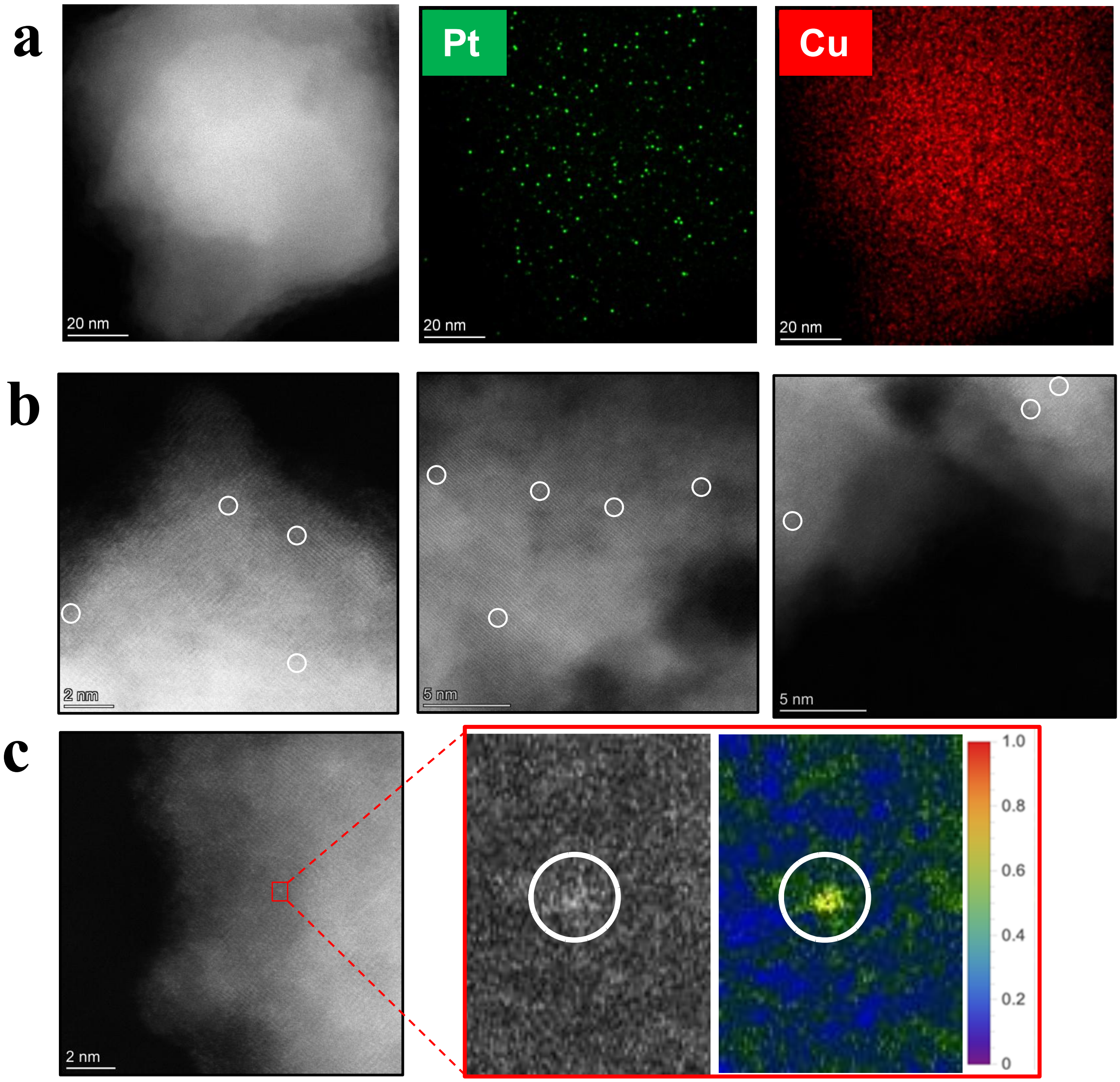

Supplementary Fig. S4: Morphology analysis of Pt_1Cu SAA. **a** STEM-EDS mapping of the Pt_1Cu SAA catalyst, showing a uniform distribution of Cu and Pt elements. **b** HAADF-STEM images of the Pt_1Cu SAA catalyst. The white circle highlights the single-dispersed Pt atom. **c** HAADF-STEM images of the Pt_1Cu SAA catalyst, the red square of the highlighted region showing isolated Pt atoms.

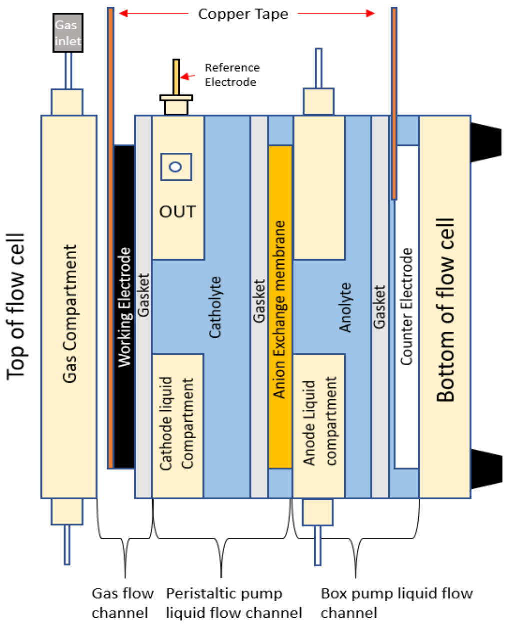

Supplementary Fig. S5: Schematic of the flow cell reactor used in this study. The reactor consists of 3 compartments – Two compartments are dedicated for continuous flow of electrolyte through external pumps and one compartment is for the gas flow channel. The schematics also shows the electrode connections using copper tapes and the places for counter and reference electrodes.

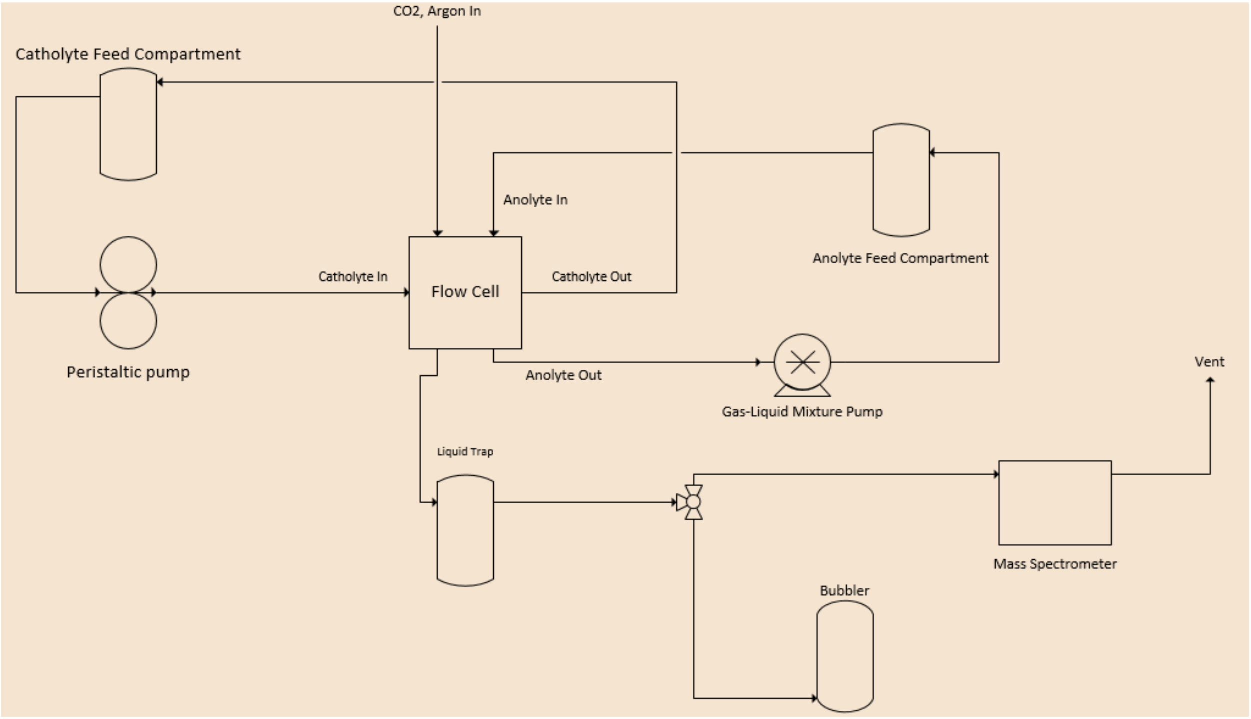

Supplementary Fig. S6: Schematic of electrochemical CO_2 reduction system experimental set-up.

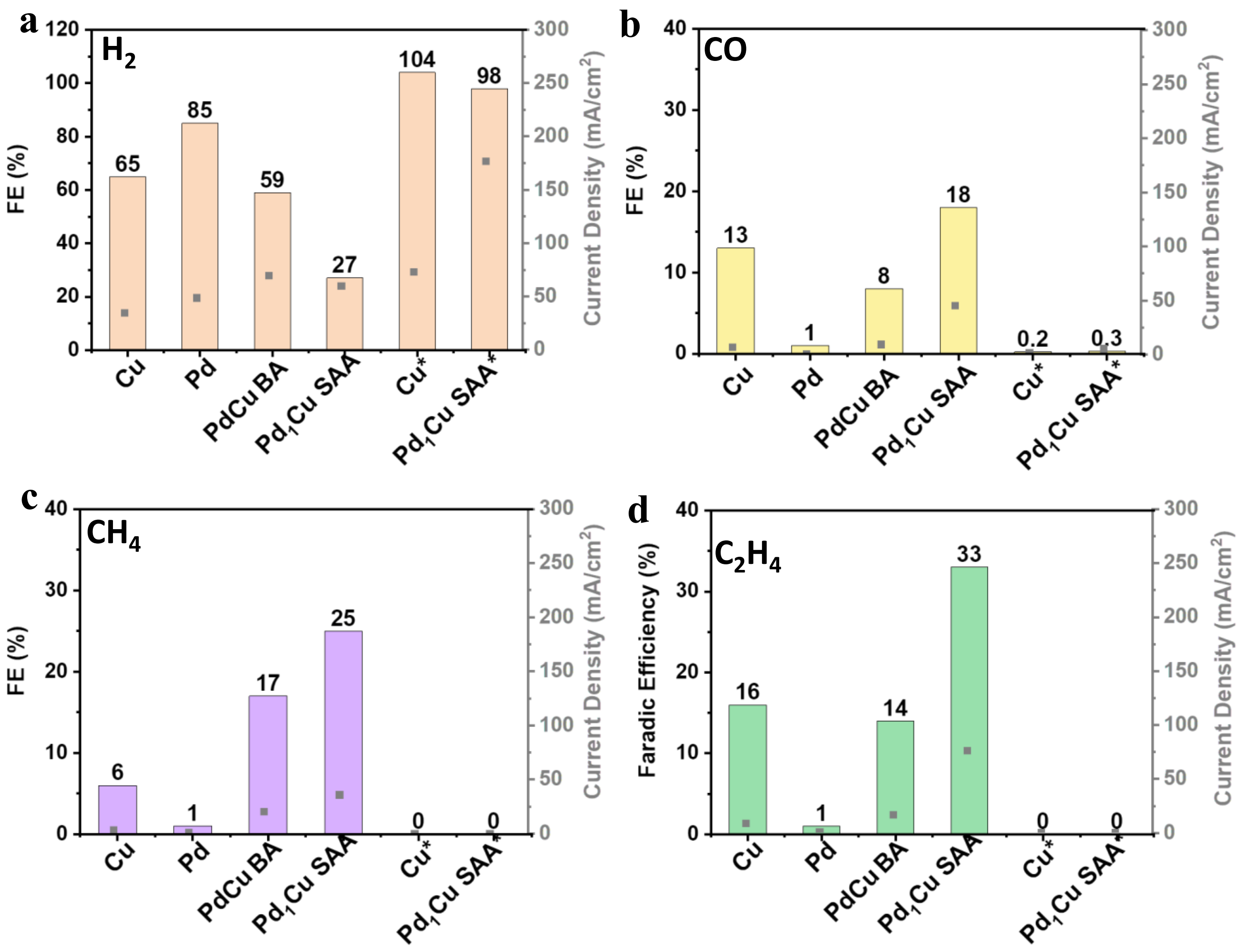

Supplementary Fig. S7: Comparisons of FE% of different products and current densities during CO_2RR at -1.1 V *vs.* RHE with Cu, Pd_1Cu single atom alloy (SAA), and PdCu bimetallic alloy (BA) polycrystalline catalysts. a Hydrogen, **b** Carbon Monoxide, **c** Methane, and d Ethylene. Cu* and Pd_1Cu SAA* represent the catalysts tested under an identical reaction setup but with Ar-only feed stream instead of CO_2. Under this non-CO_2RR condition noted with*. The catalyst loading on the GDE was maintained at 220 μg/cm^2.

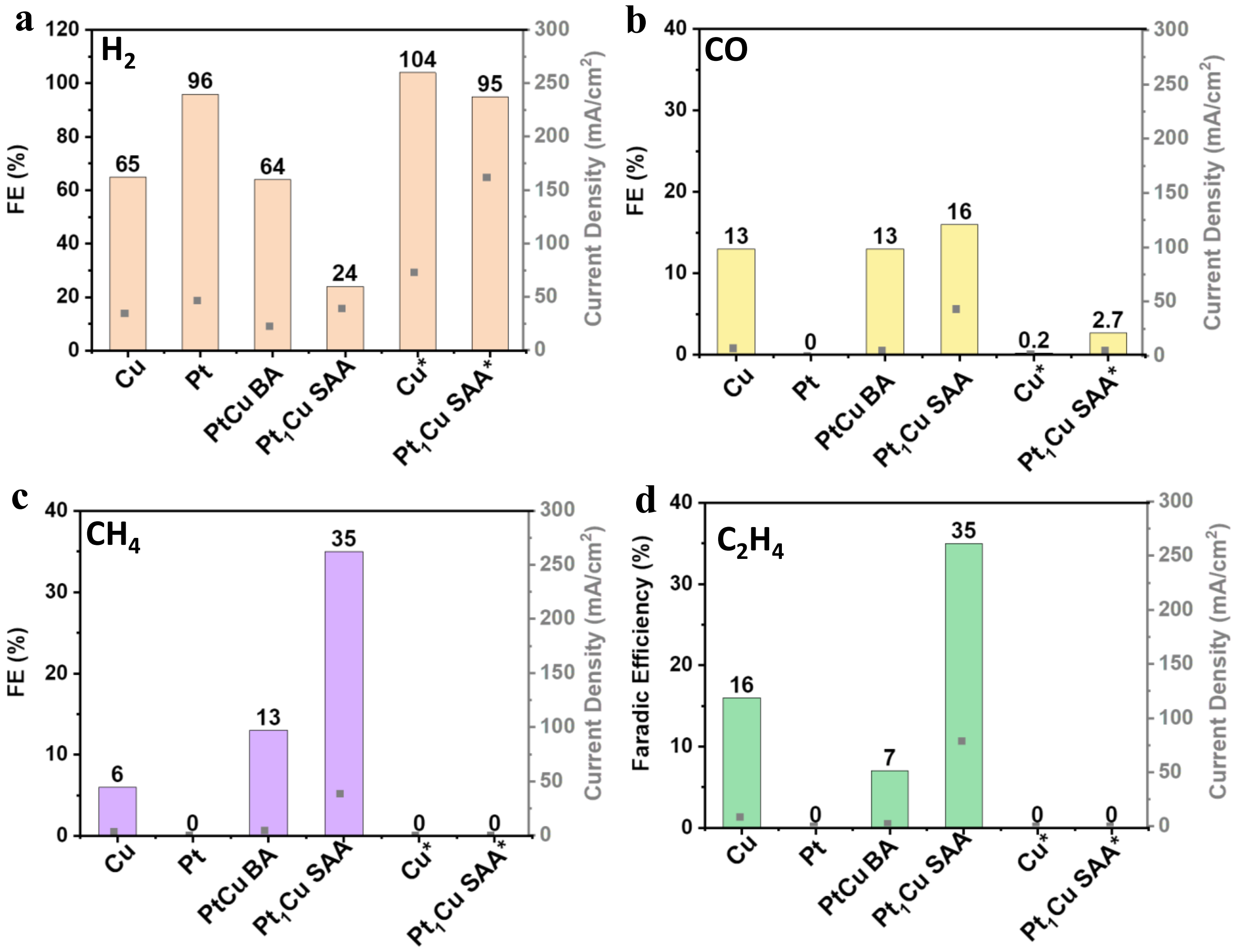

Supplementary Fig. S8: Comparisons of FE% of different products and current densities during CO_2RR at -1.1 V *vs.* RHE with Cu, Pt_1Cu single atom alloy (SAA), and PdCu bimetallic alloys (BA) as polycrystalline catalysts. a Hydrogen, **b** Carbon Monoxide, **c** Methane, and d Ethylene. Cu* and Pd_1Cu SAA* represent the catalysts tested under an identical reaction setup but with Ar-only feed stream instead of CO_2. Under this non-CO_2RR condition noted with*. The catalyst loading on the GDE was maintained at 220 μg/cm^2.

As a control experiment, we performed reaction tests with an Ar-only gas feed stream, while keeping the rest of the setups the same. H_2 was the only product that we had detected in an appreciable amount (FE% over 95 %, Supplementary Fig. S7 & Fig. S8). This validates that the hydrocarbon products during the CO_2RR originate from the gaseous CO_2 feed stream.

Supplementary Table S3: Comparison of Faradaic efficiency of liquid products obtained by CO_2 reduction using parent Cu and its BA and SAA counterparts.

Sample	Faradaic Efficiency (%)			
	Methanol	Ethanol	Formate	Acetate
Cu	1	1	2	0
CuPd BA	1	1	1	0
Pd_1Cu SAA	5	3	1	0
CuPt BA	1	1	1	1
Pt_1Cu SAA	5	2	2	0

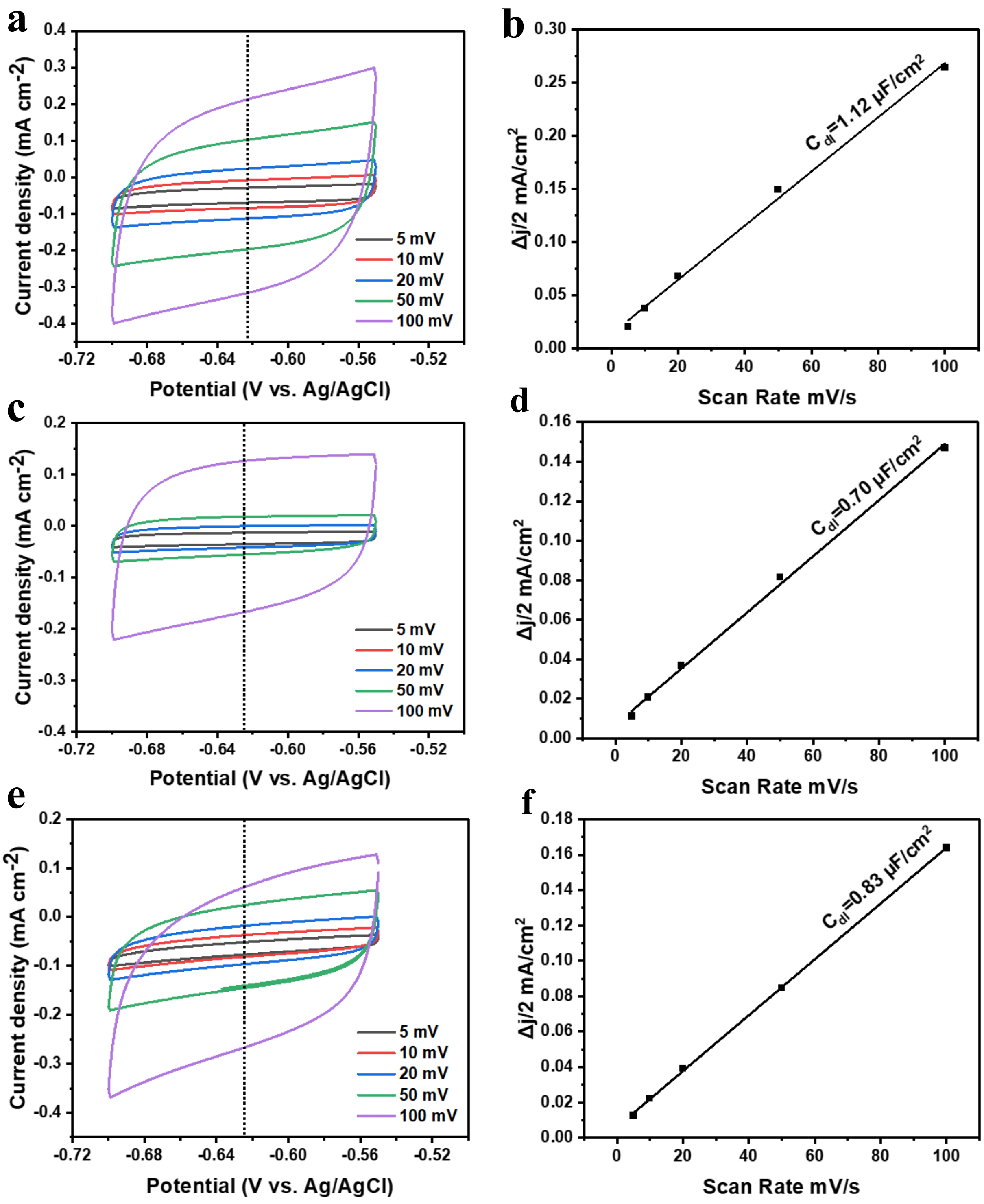

Supplementary Fig. S9: Electrochemically active surface area (ECSA) study of Cu, Pd_1Cu and Pt_1Cu. CV curves of **a** Cu, **c** Pd_1Cu, and **e** Pt_1Cu at an overpotential window of -0.55- -0.7 V *vs.* Ag/AgCl. Calculated double layer capacitance (Cdl) values of **b** Cu, **d** Pd_1Cu, and **f** Pt_1Cu. C_{dl} is equated to ECSA as discussed in section below.

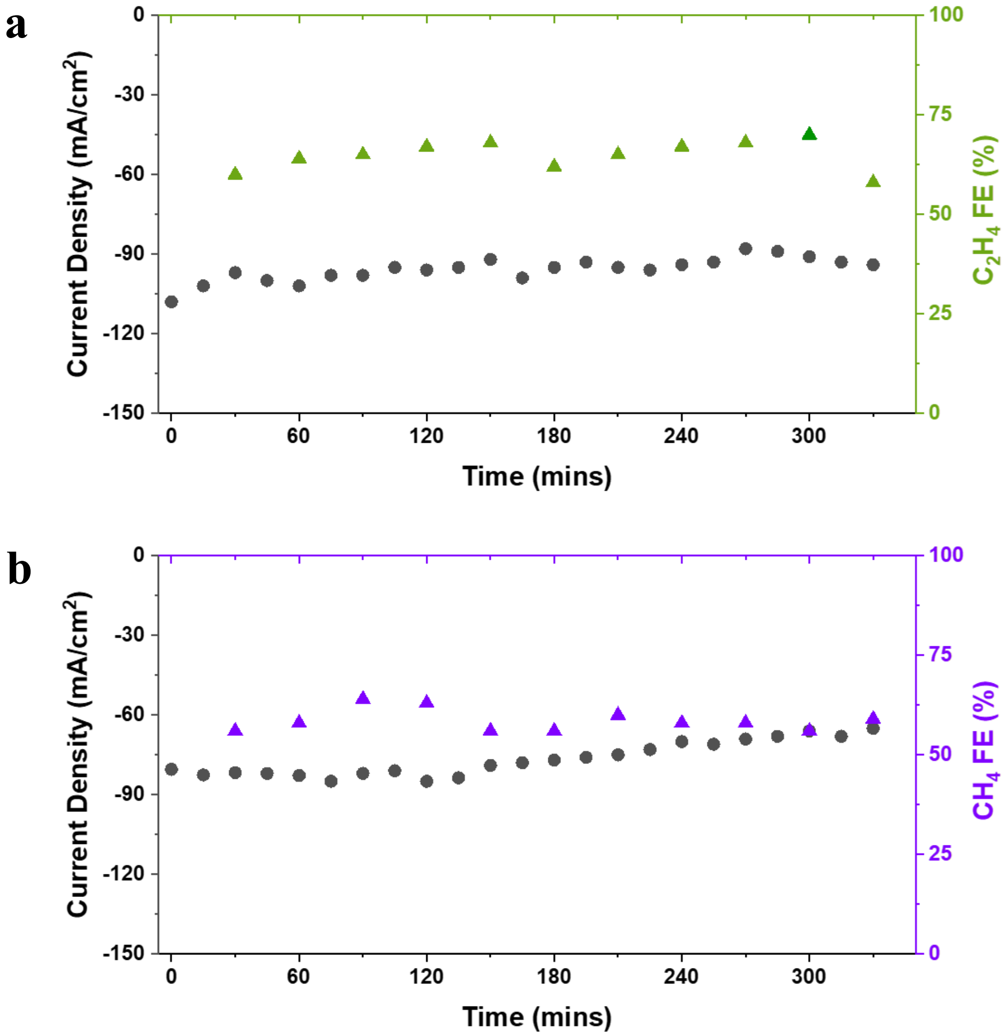

Supplementary Fig. S10: Stability test of catalysts. Amperometric *i-t* curve for **a** Cube-Pd_1Cu SAA (focusing on ethylene production) and **b** Octa-Pd_1Cu SAA (focusing on methane production) at -1.1 V *vs.* RHE. In comparison with the initial 30 min activity reported in Fig. 2, the difference in current density may be attributed to the lower catalyst loading on the GDE at around 200 μg/cm^2 for this test and the possible loss of catalyst due to washing off by electrolyte flow over time.

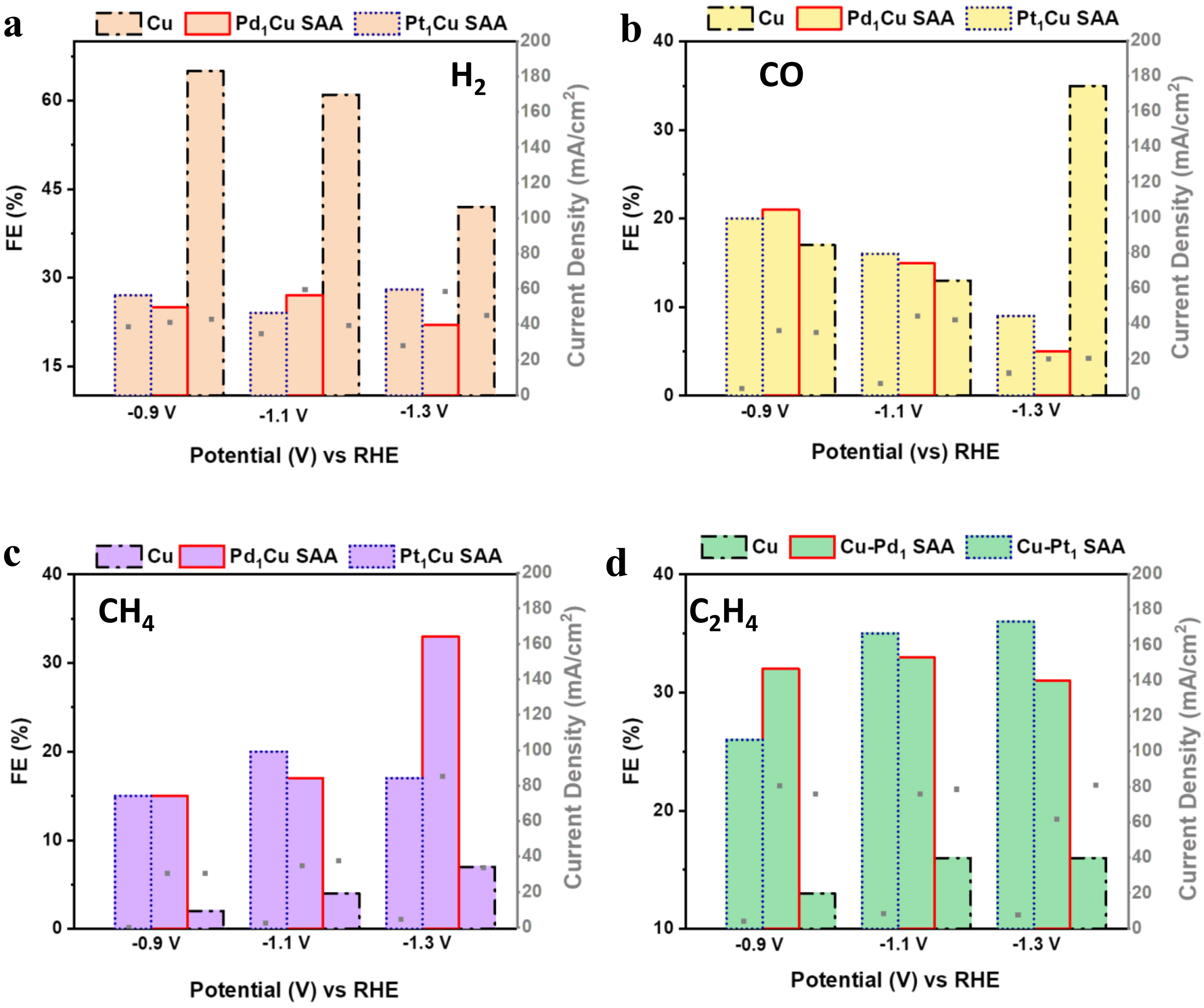

Supplementary Fig. S11: Comparisons of FE% of different products and current densities as a function of different voltage obtained with polycrystalline Cu, and M₁Cu single atom alloy (SAA) (M = Pd, Pt) as catalysts. a Hydrogen, **b** Carbon Monoxide **c** Methane, **d** Ethylene. The catalyst loading on the GDE was maintained at 220 µg/cm^2.

Supplementary Table S4: Comparison of CO_2 reduction activity of bimetallic alloys of Cu with PGM and the SAA alloys of Cu with other transition metals.

Catalyst		Products	Conditions	Performance Assay (FE)	References
Existing SAA producing CO and Formate	$Cu_{97}Sn_3$ SAA	H_2 (1)<br>CO (2)<br>Formate (3)	@ -0.7 V (RHE)<br>1 M KOH	1% (1)<br>98% (2)<br>1% (3)	2
	Pb_1Cu SAA	H_2 (1)<br>Formate (2)	@ 500 mA/cm2 current density and -0.8 V (RHE)<br><br>0.5 M $KHCO_3$,	4% (1)<br>96% (2)	3
	$Cu_{20}Sn_1$ SAA	H_2 (1)<br>Formate (2)<br>CO (3)	@ 26mA/cm^2 and -1.0 V (RHE)<br><br>H- Cell, 0.5 M $KHCO_3$, bipolar membrane	2.5% (1)<br>1% (2)<br>95.3% (3)	4
Varying ratio of PGM on Cu surface producing CO, formate and alcohols	Cu_3Pd_7	CO	@ -0.8 V (RHE)<br>H.5M $KHCO_3$.<br><br>H-Cell, Nafion 117 membrane	-80%<br>-7 mA/cm^2 at -1.2 V.<br>-7:3 is the optimal ratio of Pd:Cu<br>-With higher Cu, CO decreases and H_2 increases.	5
	Spherical Cu_3Pd<br>Flower like Pd_3Cu	CH_4<br>CO	@ -1.3 V (RHE)<br>@-1.3 V (RHE)<br>H.5M $KHCO_3$.<br>H-Cell, Nafion 115 membrane	-34% for CH_4 and 28% H_2.<br>-60% for CO and 38% for H_2.<br>- Current density of 4 mA/cm^2 for CH_4 and 13 mA/cm^2 for CO.	6
	$Pd_{56}Cu_{44}$/C	CO	@ -1.2 V (RHE)<br>H.5M $KHCO_3$.<br>H-Cell, Nafion 115 membrane	- 65%<br>-Current Density of 13 mA/cm^2 for CO and 7 mA/cm^2 for H_2.	7
	CuPd-0.3	CO	@ -0.87 V (RHE)<br>0.5 M $KHCO_3$.	-93%	8

			H-Cell, Nafion 117 membrane	-Current Density of 5.4 mA/cm^2.	
	Cu-Pd Bimetallic<br>-Ordered (1)<br>-Disordered (2)<br>-Phase Segregated (3)	CO<br>CH_4<br>C_2H_4<br>C_2H_5OH	@ -0.5 V to -1.0 V (RHE)<br>1M KOH<br>Anion exchange membrane<br>Flow Cell<br>Current density 250 mA/cm^2	-72% (1); 48% (2); 18% (3)<br>- 4% (1); 2% (2); 0.5% (3)<br>- 48% (1); 12% (2); 2% (3)<br>-15% (1); 5% (2); 1% (3)	9
	Underpotentially deposited Cu on Pd<br>UPD-CuPd	HCOOH	@ -0.15 V (RHE)<br>0.5M $NaHCO_3$.<br>Anion exchange membrane<br>H-Cell	- 84%<br>- Current density 1.8 mA/cm^2.	10
Varying ratio of PGM on Cu surface producing CO and formate and alcohols	Metallic Pd_9Cu_{91}	H_2 (1)<br>CO (2)<br>Alcohol (3)<br>Formate (4)	@ -0.95 V (RHE)<br>0.5M $KHCO_3$.<br>Nafion 117 membrane<br>H-Cell<br>@-0.65 V (RHE)	-76% (1); 1% (2); 8% (3); 2% (4)<br>Current Density 1.15 mA/cm^2.<br><br>-48% (1); 3% (2); 22% (3); 10% (4)	11
	CuPd nanoparticles /C<br>Cu:Pd ratio = 1.48<br>Metal loading = 19.4 wt%.	H_2 (1)<br>CO (2)	@ -0.9 V (RHE)<br>0.1M $KHCO_3$.<br>Anion exchange membrane<br>H-Cell<br>@-1.1 V (RHE)	-13% (1); 87% (2)<br><br>Mass activity of 47 mA/mg Pd<br><br>-75% (1); 13% (2)	12
	Cu_8Pd_2 nanoalloys.	H_2 (1)<br>CO (2)<br>C_2H_4 (3)<br>Formate (4)	@ -1.07 V (RHE)<br>$KHCO_3$.<br>Nafion 115 membrane<br>H-Cell	-52 % (1); 40% (2); 3% (3); 5% (4)<br>2 mA/cm^2 current density for CO at -0.87 V (RHE)	13
	Pd decorated Cu	H_2 (1)<br>CO (2)<br>CH_4 (3)<br>C_2H_4 (4)	@ -0.96 V (RHE)<br>0.5 M $KHCO_3$.<br>Anion exchange membrane<br>H-Cell	-45% (1); 1% (2); 46% (3); 8% (4)	14

				Current Density<br>60mA/cm^2 at -<br>0.96 V	
	$Cu_{85}Pt_{15}$<br>alloy<br>nanocubes	H_2 (1)<br>CO (2)	@ 1.75 V (Ag/AgCl)<br>0.5 M $KHCO_3$.<br>Nafion 117 membrane<br>H-Cell	-75% (1); 25%<br>(2)	15
Promising<br>Dilute PGM-Cu<br>alloys for<br>hydrocarbon<br>production	CuPd<br>Pentagonal<br>bipyramids	H_2 (1)<br>CH_4 (2)<br>C_2H_4 (3)<br>Alcohol (4)<br>Formate<br>(5)	@ -1.0 V (RHE)<br>0.5M $KHCO_3$.<br>Anion exchange membrane<br>H-Cell	-18% (1); 5%<br>(2); 31% (3);<br>19% (4); 11%<br>(5)<br>Current Density<br>35 mA/cm^2.	16
	(a) Cu_3Pt<br>(b) Cu_5Pt_1<br>(Increasing<br>the Cu:Pt<br>ratio<br>decreases the<br>H_2 FE)	H_2 (1)<br>CH_4 (2)	@ -1.35 V (SCE)<br>0.5 M $KHCO_3$.<br>Nafion 117 membrane<br>H-Cell	(a) 10% (1);<br>22%<br><br>(b) 7% (1); 12%<br>(2)	17
This work:<br>Enhancing<br>hydrogenation<br>by PGM SAA<br>on Copper<br>surface	Cu<br>polycrystalli<br>ne	H_2 (1)<br>CO (2)<br>CH_4 (3)<br>C_2H_4 (4)	@ -0.9 V (RHE)<br><br>0.5 M $KHCO_3$<br><br>Flow cell<br><br>~200 mA/cm^2 current density	65% (1)<br>20% (2)<br>2% (3)<br>13% (4)	This<br>Work
	Pd_1Cu SAA			25% (1)<br>21% (2)<br>20 (3)<br>26% (4)	
	Pt_1Cu SAA			27% (1)<br>17% (2)<br>20% (3)<br>32% (4)	
This work:<br>Enhancing<br>hydrogenation<br>by PGM SAA<br>on Copper<br>surface	Pd_1Cu SAA<br>(With Cu<br>octahedra)	H_2 (1)<br>CO (2)<br>CH_4 (3)<br>C_2H_4 (4)	@ -0.9 V (RHE)<br><br>0.5 M $KHCO_3$<br><br>Flow cell<br><br>~220 mA/cm^2 current density	17% (1)<br>1% (2)<br>60% (3)<br>21% (4)	This<br>Work
	Pd_1Cu SAA<br>(With Cu<br>cube)			25% (1)<br>3% (2)<br>16% (3)<br>55% (4)	

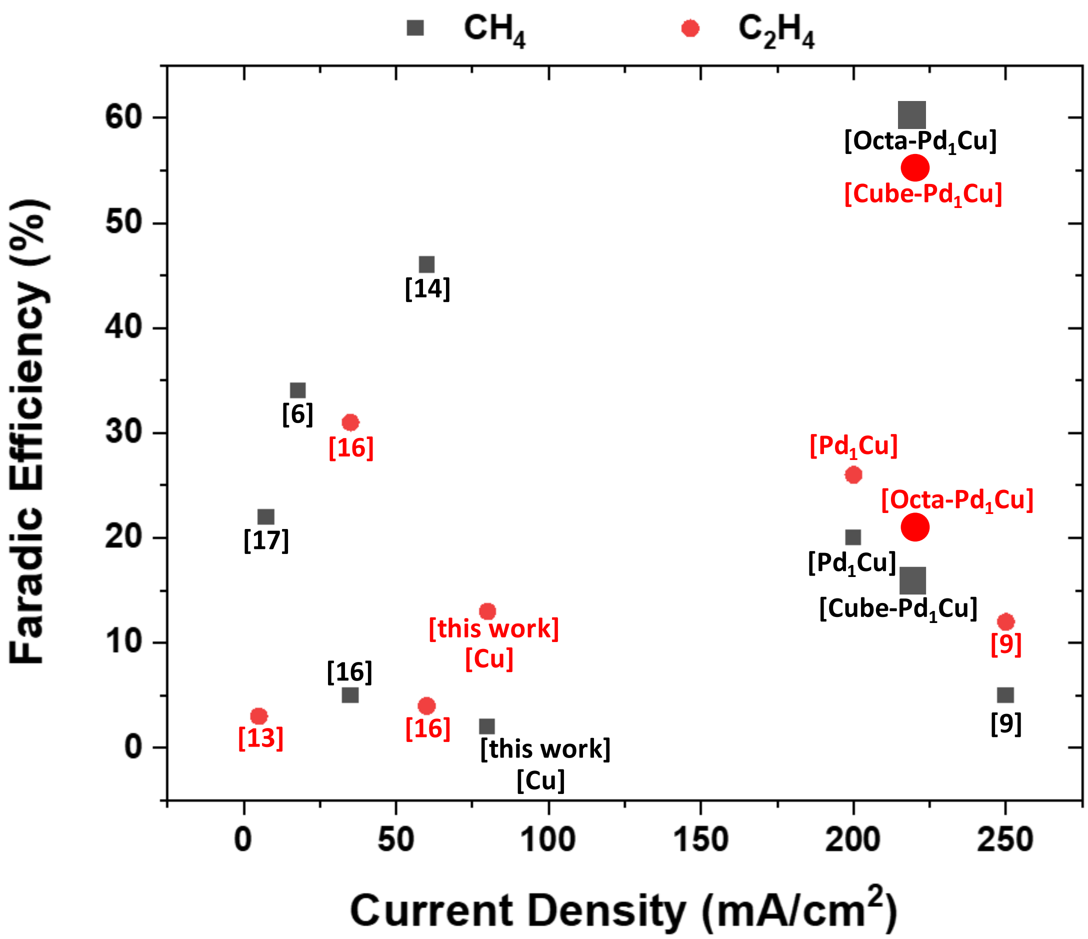

Supplementary Fig. S12a: CH_4 or C_2H_4 FE% and corresponding partial current density comparison of various transition metal-copper bimetallic catalysts with the catalyst developed in this work. The detailed experimental parameters and conditions for each data can be seen in Table S4. Readers are cautioned to consider that the present study is not aimed at showing the absolute best catalytic activity. The activity obtained here is not the best among all possible reaction setup configurations to make hydrocarbon molecules from CO_2RR. Through the present study, we focus on proving the hypothesis-driven results that atomically dispersed PGMs on copper surfaces will improve the CO_2RR efficiency toward CO_2 hydrogenation reactions, which is otherwise difficult to achieve by using catalysts with extended PGM surfaces or alloying copper with other transition metals.

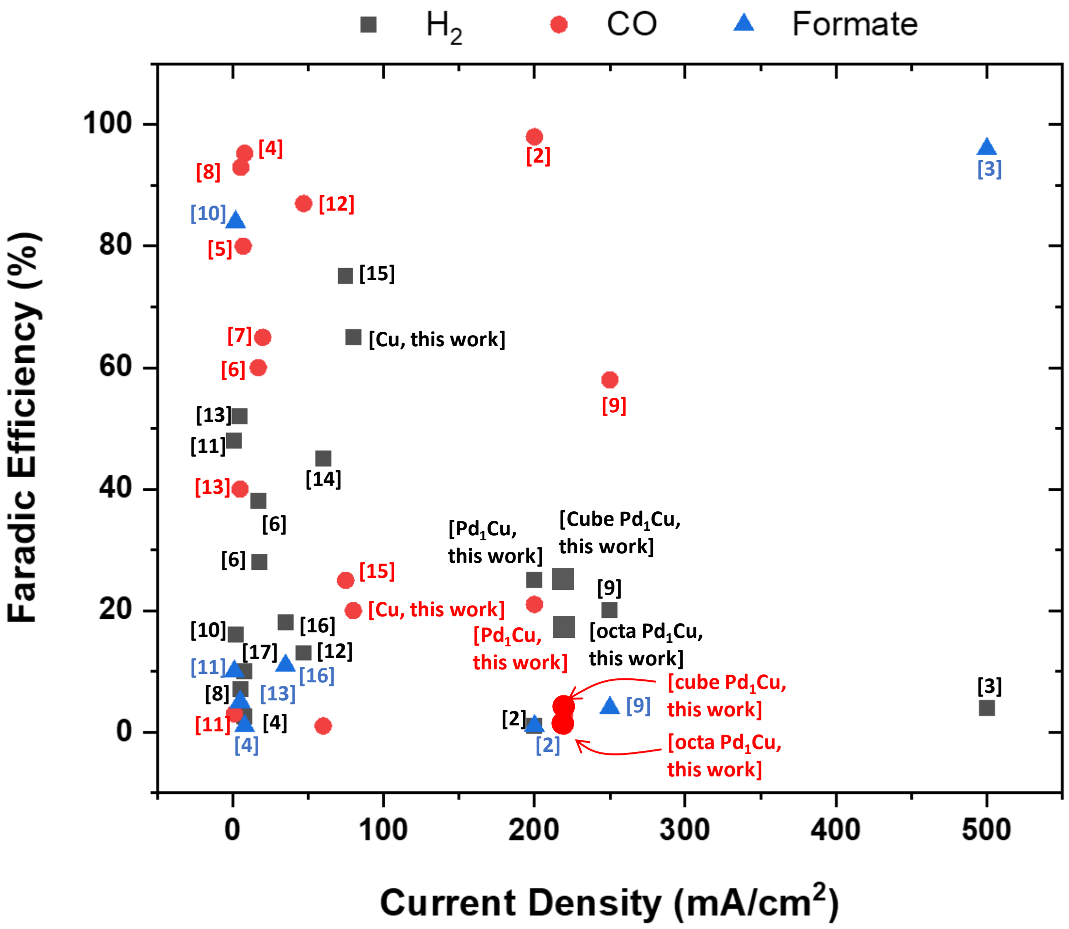

Supplementary Fig. S12b: FE% and corresponding partial current density comparison for representative non-hydrocarbon products (undesired in this work) during CO_2RR for various transition metal-copper bimetallic Cu catalysts in comparison with the catalysts developed in this work. The detailed experimental parameters and conditions for each data can be seen in Supplementary Table S4 in supporting information. Readers are cautioned to consider that the present study is not aimed at showing the absolute best catalytic activity. The activity obtained here is not the best among all possible reaction setup configurations to make hydrocarbon molecules from CO_2RR. Through the present study, we focus on proving the hypothesis-driven results that atomically dispersed PGMs on copper surfaces will improve the CO_2RR efficiency toward CO_2 hydrogenation reactions, which is otherwise difficult to achieve by using catalysts with extended PGM surfaces or alloying copper with other transition metals.

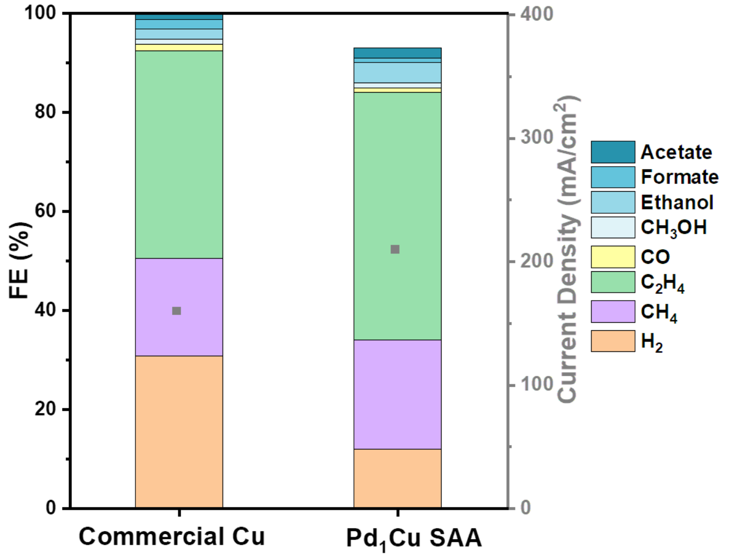

Supplementary Fig. S13a: Comparison of CO_2RR FE% and current densities for Commercial Cu and Pd_1Cu SAA. (The catalyst loading on the GDE was maintained at 1 mg/cm^2, and the FE% analyses were performed by running at -1.1 V *vs*. RHE in 0.5 M $KHCO_3$.)

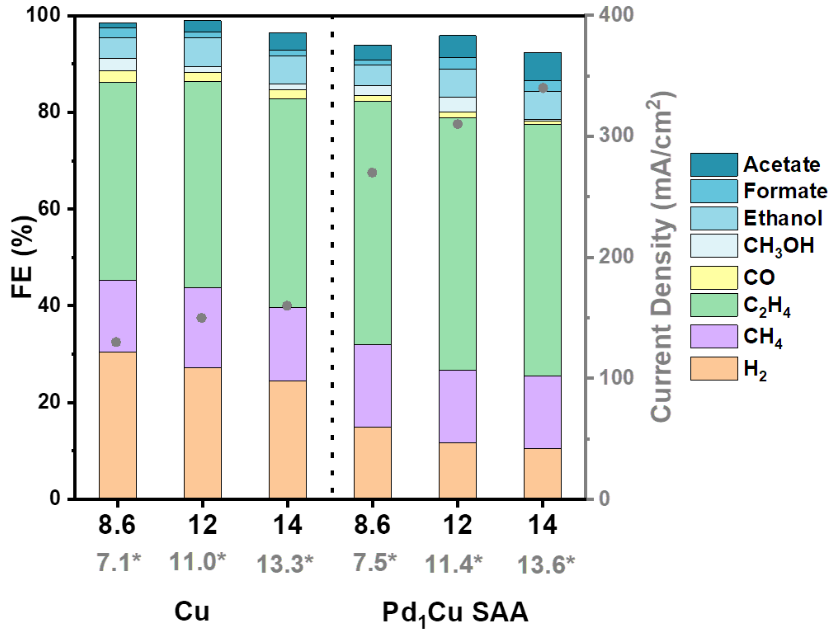

Supplementary Fig. S13b: Comparison of CO_2RR FE% for various products and current densities under various electrolyte pHs at −1.1 V *vs.* SHE from Cu and Pd_1Cu SAA catalysts. (The catalyst loading on the GDE was maintained at 1 mg/cm^2, Electrolytes: 1.0 M KOH (pH 14), 0.5 M K_2CO_3 (pH 12), 1.0 M $KHCO_3$ (pH 8.6), the surface pHs donated as * were estimated using the approaches reported in recent literature [18-20].) The narrowed reactivity difference between the catalysts in this plot compared with Fig. 2 was caused by the c.a. 450 % higher catalyst loading, where the mass transfer limitations became more significant during the reaction.

To further decouple the positive impacts to the targeted reaction due to the alloy catalysis and the consequential increasing pH due to faster proton consumption:

We first focused on how each catalyst changes its performance under different pHs by examining its activity in different electrolytes. The results revealed that the current densities of both parent Cu and Pd_1Cu SAA catalysts increased when the pH increased from 8.6 to 14. Regarding reaction activity, H_2 formation rates (partial current densities) for both catalysts remained relatively constant or across different pH conditions. For the gaseous hydrocarbons, especially the C_2H_4, the higher pHs led to a c.a. 30% enhancement in the C_2H_4 production rate on both catalysts. This improvement can be attributed to the enrichment of OH^- species that lowered CO binding energy on the Cu surface and reduced the activation energy barrier for the CO dimerization step to C_{2+} formation, according to the literature [20-23]. In short, the increases in pH, either as the result of in-situ proton consumption or manually increased bulk pH, will lead to reaction performances favoring the CO_2 hydrogenation against HER.

Then, we proceeded to compare the activities between the Cu and SAA catalysts. Upon introducing Pd single atoms, the current density exhibited a c.a. 100% increase compared to the parent Cu. Regarding specific product activity, the H_2 production rate for the SAA was akin to that observed for Cu, suggesting that introducing Pd single atoms did not enhance HER. When comparing the SAA with its parent Cu across each pH value we investigated, the CH_4 and C_2H_4 production rates increased by c.a.100 % and at least 150 %, respectively. We speculate that the presence of Pd single atoms facilitated the CO_2 hydrogenation beyond the pH promotion effect because such an enhancement is still apparent when comparing any higher-pH Cu catalyst with a lower-pH SAA catalyst. To further decouple intrinsic Pd-Cu chemistry and the pH effect, we estimated surface pHs at the catalyst surfaces (denoted as * in Fig. S13b) under our reaction conditions by referring to the reported models and methodologies [18-20], which arguably provide more realistic pH values near the catalyst surfaces. Therefore, the local pH increase caused by high current density indeed promotes CO_2RR to some extent. Still, the single-atom alloy (SAA) of PGMs is a more significant contributor.

Electrochemically Active Surface area:

The Electrochemically Active Surface Area (ECSA) was estimated by the electrochemical double layer (ECDL) capacitance of the electrode[24,25]. The double layer capacitance is measured by CV method in the non-Faradaic region (i.e. potential range between -0.55 V to -0.70 V). The difference between the cathodic (ic) and anodic (ia) sweeps at a given voltage scales linearly with the scan rate (v), and the slope of the line is equal to twice the double-layer capacitance C_{dl}.

$$2 \times C_{dl} = \frac{i_c - i_a}{v}$$

The galvanic displacement reaction of PGM precursor solution with metallic Cu NPs may introduce surface etching and corrugation on Cu surface. This may increase the surface area of the PGM_1Cu SAA catalyst in comparison to the parent Cu catalyst, which in turn may also improve the overall catalytic activity. Therefore, we estimated these catalysts' electrochemically active surface area (ECSA) by cyclic voltammogram in the non-Faradaic region to measure the double-layer capacitance. Since the double-

layer capacitance is directly proportional to ECSA, we could compare the ECSA value of the catalysts. We find that Pd_1Cu and Pt_1Cu SAAs have similar ECSA, trailing behind the ECSA of the parent Cu NPs (Supplementary Fig. S9). Thus, we conclude that introducing PGM single atoms on the Cu surface does not necessarily improve the ECSA, even though the geometrical surface area may be changed due to etching. This suggests that the activity improvement caused by the SAA strategy in this work is mainly due to the chemistry modification instead of any increased ECSA.

Computational Setups:

All density functional theory (DFT) calculations were carried out using Vienna *Ab Initio* Simulation Package (VASP) code[26,27]. To treat exchange-correlation interactions in a periodic boundary system, we applied the revised Perdew-Burke-Ernzerhof (RPBE) functional[28] and the projector augmented wave (PAW) method[29]. A Monkhorst-Pack mesh[30] k-point grids of (3×3×1) and an energy cutoff for the valence plane waves of 400 eV were used in this work. To model the Pd_1Cu catalysts, we chose the CO_2RR on Cu(100) surface and on Cu(111) surfaces and exchanged a single Cu atom with a single Pd atom on the surfaces (Supplementary Fig. S14). The supercell size was p(4×4) and the four-layers Cu slab was constructed with the two bottom layers fixed to its bulk location and the two upper layers relaxed. All the examined surfaces have a ~15 Å vacuum layer separation between each periodic unit cell to avoid z-direction lateral interaction. The lattice constant of Cu was calculated as 3.68 Å, which is similar with previous result[31]. Geometries were considered optimized when the energy had converged to 10^{-5} eV and the forces were smaller than 0.03 eV/Å. To search for the transition state (TS), we used CI-NEB method[32] to get a better initial guess for the minimum energy pathway and used Dimer method[33] to find the true TS. The true TS was further verified by calculating their vibrational frequencies (ν_i) confirming that a single imaginary frequency was obtained which corresponds to the negative curvature at the saddle point.

The adsorption energy (E_{ad}) can be calculated according to Eqn. (S1),

$$E_{ad} = E_{total} - E_{surface} - E_{adsorbate} \tag{S1}$$

where E_{total} represents the energy of species over surface, $E_{surface}$ represents the energy of the surface and $E_{adsorbate}$ represents the energy of adsorbate in the gas phase. The reaction energy (ΔH_{rxn}) can be calculated according to Eqn. (S2),

$$\Delta H_{rxn} = E_{FS} - E_{IS} \tag{S2}$$

where E_{FS} represents the energy of the final state (FS) and E_{IS} represents the energy of initial state (IS). The activation barrier ($\Delta E^{‡}$) can be calculated according to Eqn. (S3),

$$\Delta E^{‡} = E_{TS} - E_{IS} \tag{S3}$$

where E_{TS} represents the energy of the TS.

All the energies from DFT calculations (E) were further corrected by zero-point energy (ZPE) and entropy (S) to obtain free energy (G) shown in Eqn. (S4). The equation of ZPE for each examined specie is given by Eqn. (S5). Since translational and rotational degree of freedom for surface adsorbates become restricted translational- and rotational-vibrational modes, only vibrational entropy contribution (S_{vib}) was

considered for the entropy correction for surface species (Eqn. (S6)). The vibrational frequencies (ν_i) for surface species were calculated using DFT simulations. When we calculated the vibrational frequency of adsorbates, we have fixed all atoms in the slab and only relaxed the adsorbates to avoid the vibrational frequency noises from the surface. The entropy for gas phase molecule at room temperature ($T = 298.15$ K) and standard pressure (1 bar) can be referred to JANAF thermochemical table[34].

$$G = E + ZPE - TS \quad (S4)$$

$$ZPE = \frac{1}{2}\sum_i h\nu_i \quad (S5)$$

$$S_{vib} = R\sum_i[\frac{h\nu_i}{k_B T}\frac{e^{-\frac{h\nu_i}{k_B T}}}{1-e^{h\nu_i}} - \ln(1 - e^{-\frac{h\nu_i}{k_B T}})] \quad (S6)$$

where k_B is Boltzmann constant and R is gas constant.

For hydrogen revolution reaction (HER) calculation, we applied computational standard hydrogen electrode model[35], which allows us to approach the energy of a pair of protons (H^+) and electron (e^-) to the one of half of hydrogen molecule in the gas phase ($\frac{1}{2}H_2$) at standard condition (P = 1 bar, T = 298.15 K and pH = 0) under U_{SHE} = 0 V shown in Eqn. (S7),

$$H^+ + e^- \leftrightarrow \frac{1}{2}H_2 \quad (S7)$$

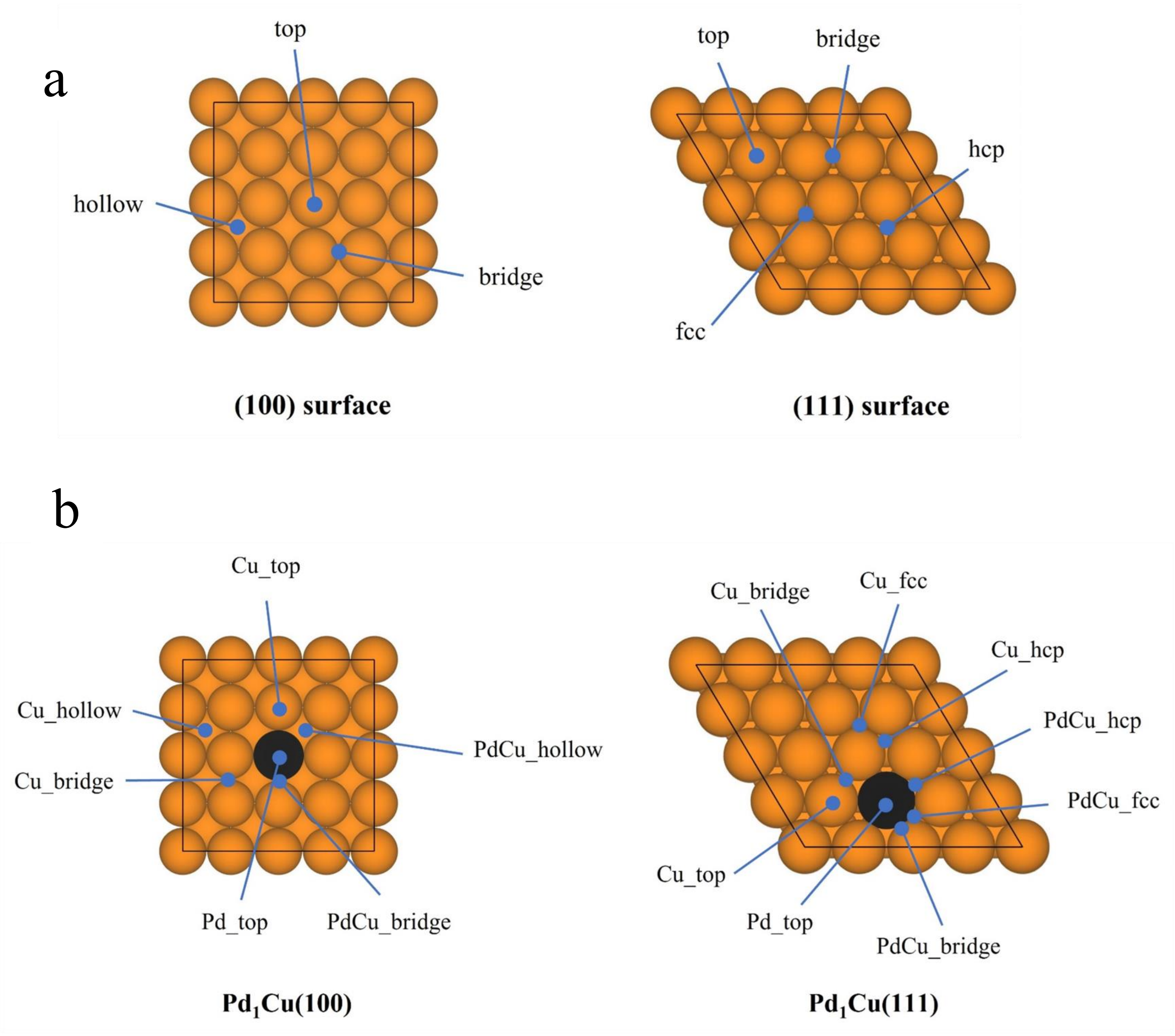

Supplementary Fig. S14: The possible adsorption sites over the catalysts. a The possible adsorption sites over the pure Cu (100) and (111) surfaces. **b** The possible adsorption sites over the $Pd_1Cu(100)$ and $Pd_1Cu(111)$ surfaces. To investigate the role of single Pd atom on the Pd_1Cu catalyst, we have examined the CO_2RR to C_1 or C_2 products over pure Cu and Pd catalysts for their (100) and (111) surfaces and compared the energetics among pure Cu, pure Pd, and Pd_1Cu catalysts.

Supplementary Table S5: Summary of the adsorption energies of CO* over the (100) surfaces.

Surface	Adsorption site	E_{ad} (eV)
Cu(100)	**top**	**-0.59**
	bridge	-0.55
	hollow	-0.49
$Pd_1Cu(100)$	Cu_top	-0.57
	Cu_bridge	-0.60
	Cu_hollow	-0.54
	**Pd_top**	**-1.02**
	PdCu_bridge	-0.91
	PdCu_hollow	-0.79
Pd(100)	top	-1.21
	**bridge**	**-1.59**
	hollow	-1.44
$Pt_1Cu(100)$	Cu_top	-0.57
	Cu_bridge	-0.53
	Cu_hollow	-0.53
	**Pt_top**	**-1.42**
Pt(100)	top	-1.69
	**bridge**	**-1.78**

Note: CO* at the PtCu_bridge, and PtCu_hollow sites of $Pt_1Cu(100)$ is unstable and shifts to the nearby Pt_top site. CO* at the hollow site of Pt(100) is unstable and shifts to the nearby bridge site. The most favorable adsorption site for CO* over each (100) surface is bolded.

Supplementary Table S6: Summary of the adsorption energies of CO^* over the (111) surfaces.

Surface	Adsorption site	E_{ad} (eV)
Cu(111)	top	-0.51
	bridge	-0.50
	**fcc**	**-0.54**
	**hcp**	**-0.54**
$Pd_1Cu(111)$	Cu_top	-0.26
	Cu_bridge	-0.27
	Cu_fcc	-0.30
	Cu_hcp	-0.27
	**Pd_top**	**-0.73**
	PdCu_bridge	-0.51
	PdCu_fcc	-0.54
	PdCu_hcp	-0.53
Pd(111)	top	-1.13
	bridge	-1.70
	**fcc**	**-1.71**
	hcp	-1.68
$Pt_1Cu(111)$	Cu_top	-0.18
	Cu_bridge	-0.27
	Cu_fcc	-0.23
	Cu_hcp	-0.22
	**Pt_top**	**-1.06**
Pt(111)	top	-1.39
	bridge	-1.44
	**fcc**	**-1.48**
	hcp	-1.45

Note: CO^* at the PtCu_bridge, PtCu_fcc and PtCu_hcp sites of $Pt_1Cu(111)$ is unstable and shifts to the Pt_top site. The most favorable adsorption site for CO^* over each (111) surface is bolded

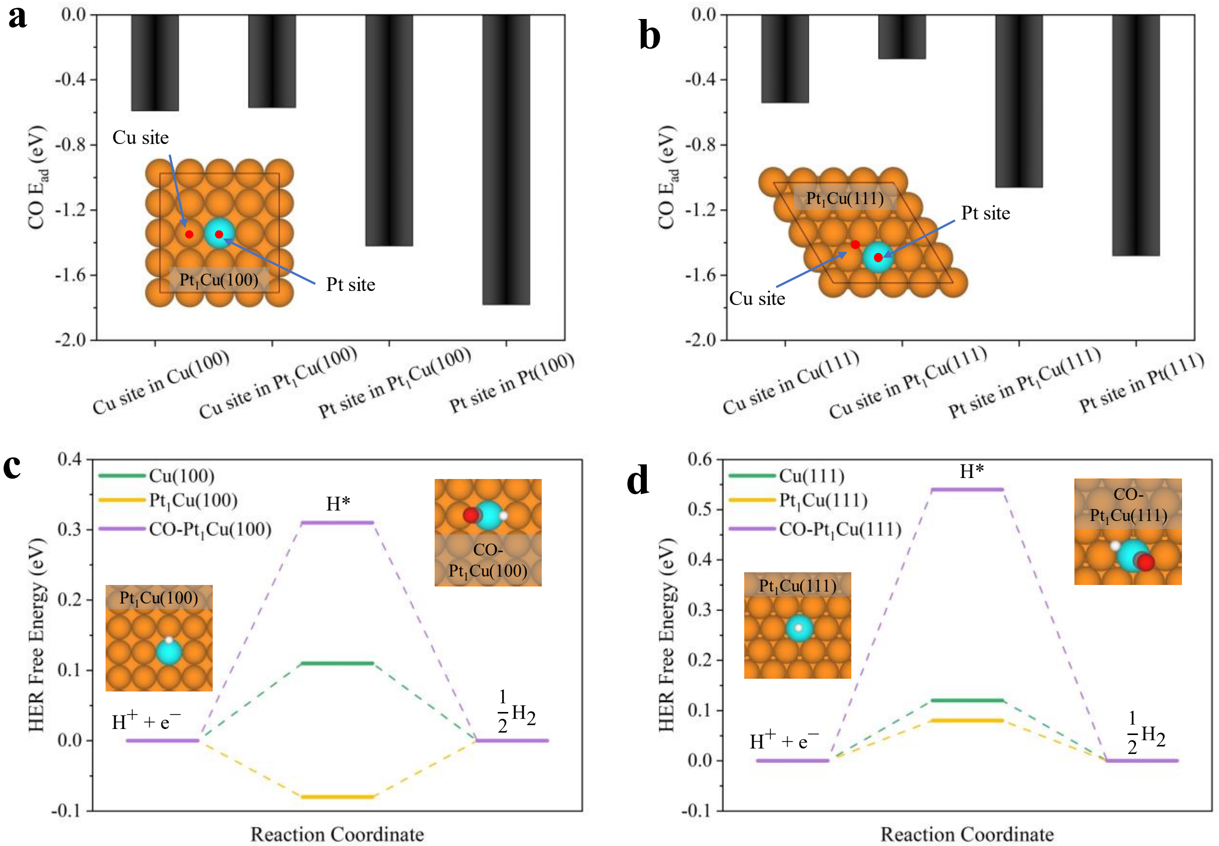

Supplementary Fig. S15: The adsorption energies of CO* and H*. **a** and **b** are the adsorption energies of CO^* over (100) and (111) facets of bare Cu, Pt_1Cu, and bare Pt, respectively. The top views of $Pt_1Cu(100)$ and (111) with their possible CO^* adsorption sites (red) are inserted. **c** and **d** are the free energy diagram of HER over (100) and (111) facets of bare Cu, Pt_1Cu, and Pt_1Cu with the presence of a CO molecule co-adsorption over the Pt site (CO-Pt_1Cu). The configurations of H^* on Pt_1Cu and CO-Pt_1Cu are inserted. Note: although the most favorable site for H^* on $Pt_1Cu(111)$ is the Pt_top site, the strong-bonded CO^* would repel H^* to its second favorable PtCu_hcp adsorption site.

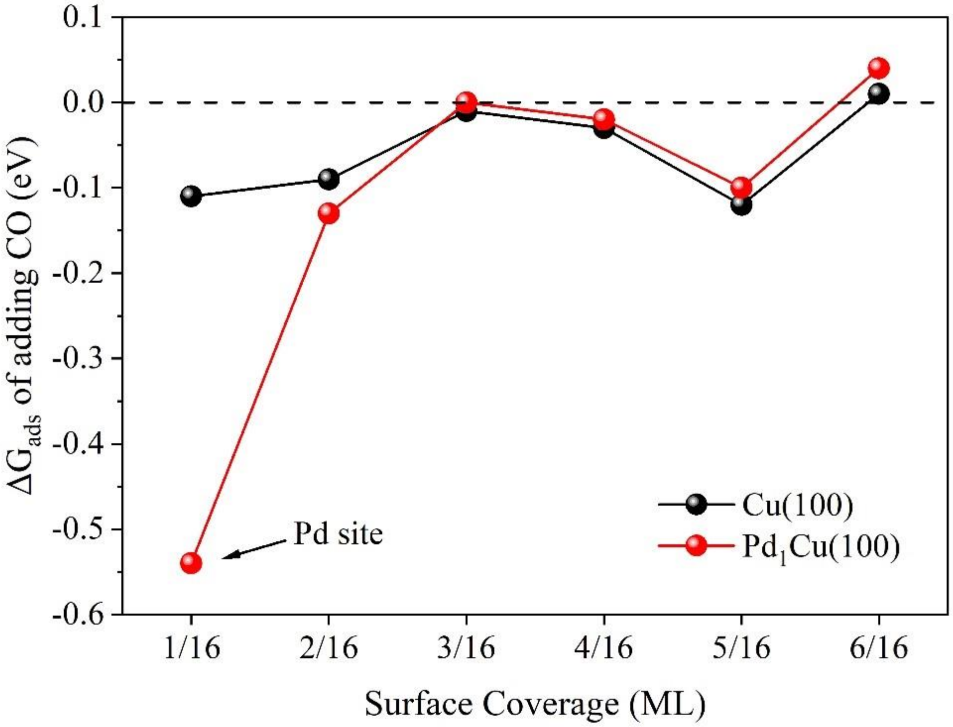

Supplementary Fig. S16: The adsorption free energy of adding CO on the Cu(100) and Pd_1Cu(100). The more negative value refers to stronger adsorption of CO.

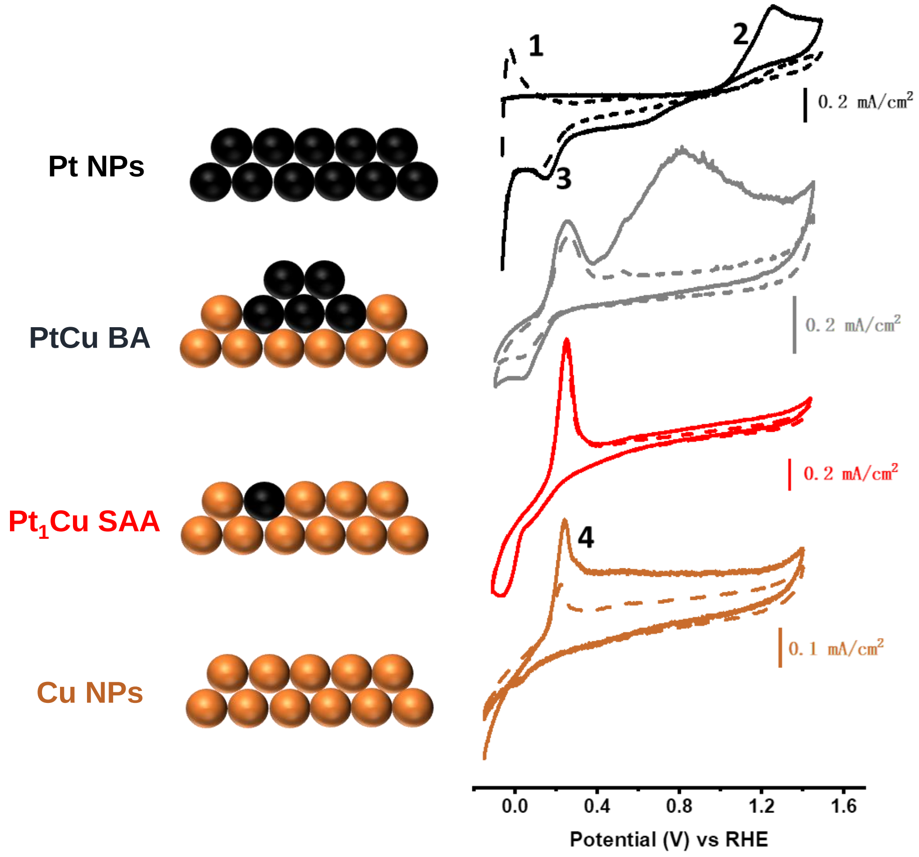

Supplementary Fig. S17: CO stripping study with various samples to investigate the CO-poisoning effect on PGM of catalyst surface during CO_2 reduction. The solid lines are the 1st cycle of CV and the dotted lines are the 2nd cycle. The peak between 0.8-1.1 V is due to CO-stripping in the first cycle. Before the experiment, CO was allowed to adsorb on the surface of the catalyst by saturating the electrolyte (0.1 M H_2SO_4) and applying 0.1 V (vs RHE) for 5 minutes. after adsorption, the electrolyte was re-purged with N_2 to remove all dissolved CO before CO stripping experiment was performed.

Cu NPs and Pt_1Cu SAA did not show any CO stripping peak (between 0.6 - 1.0 V *vs.* RHE) in the first cycle after the CO was potentiostatically adsorbed at 0.1 V for 5 mins and let alone the similar second cycle where any possible remaining -CO had already desorbed[36]. In contrast, in the second CV cycle, as marked by dashed lines for Pt-only and PtCu BA catalysts, we see a significant change of line shapes against the first cycle results, pointing towards the disappearance of the CO stripping peak and confirming the significant CO poisoning effect on these “inferior” PGM-containing catalysts in the first testing cycle. Thus, it can be concluded that Cu and Pt_1Cu SAA are not deactivated by CO^* during CO_2R process, where CO^* is one of the early reactive intermediates formed in CO_2RR steps.

Supplementary Table S7: Summary of the adsorption energies of H^* over the (100) surfaces.

Surface	Adsorption site	E_{ad} (eV)
Cu(100)	top	0.50
	bridge	0.05
	**hollow**	**0.03**
$Pd_1Cu(100)$	Cu_bridge	0.04
	Cu_hollow	-0.02
	Pd_top	0.25
	PdCu_bridge	-0.09
	**PdCu_hollow**	**-0.12**
$Pt_1Cu(100)$	Cu_top	0.54
	Cu_bridge	0.08
	Cu_hollow	-0.02
	Pt_top	-0.23
	**PtCu_bridge**	**-0.26**
	PtCu_hollow	-0.19

Note: H^* at the Cu_top site of $Pd_1Cu(100)$ is unstable and shifts to the nearby PdCu_bridge site. The most favorable adsorption site for H^* over each (100) surface is bolded. We referred to the energy of one H atom as half of the energy of H_2 in the gas phase.

Supplementary Table S8: Summary of the adsorption energy of H^* over the (111) surfaces.

Surface	Adsorption site	E_{ad} (eV)
Cu(111)	bridge	0.07
	fcc	-0.05
	**hcp**	**-0.06**
$Pd_1Cu(111)$	Cu_fcc	0.07
	Cu_hcp	0.08
	Pd_top	0.30
	**PdCu_hcp**	**-0.02**
	PdCu_fcc	0.00
$Pt_1Cu(111)$	Cu_fcc	0.09
	Cu_hcp	0.13
	**Pd_top**	**-0.11**
	PdCu_hcp	-0.07
	PdCu_fcc	-0.04

Note: H^* at the top site of Cu(111) is unstable and shifts to the hcp site; H^* at the Cu_top, Cu_bridge, and PdCu_bridge sites of $Pd_1Cu(111)$ is unstable and shifts to the PdCu_hcp site; H^* at the Cu_top and Cu_bridge sites of $Pt_1Cu(111)$ is unstable and shifts to the Cu_fcc and PdCu_hcp sites, respectively. The most favorable adsorption site for H^* over each (111) surface is bolded. We referred to the energy of one H atom as half of the energy of H_2 in the gas phase.

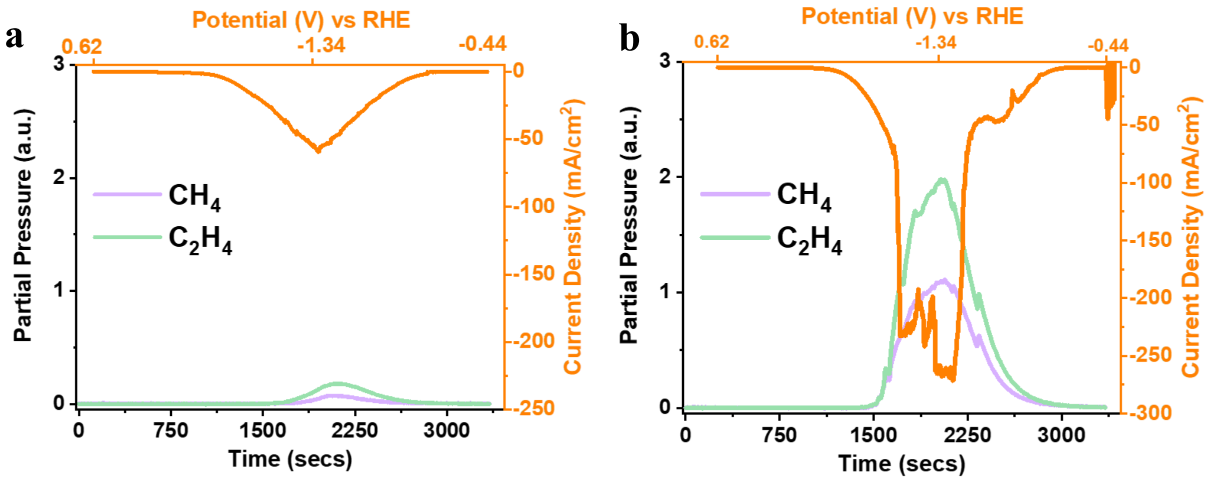

Supplementary Fig. S18: Real-time analyses of gaseous products as a function of scanning potential. Cyclic voltammograms and real-time hydrocarbon products detected by mass spectrometer during CO_2RR using **a** parent Cu NPs and **b** Pt_1Cu SAA polycrystalline catalysts. The potential was scanned at 1mV/sec, and mass spectrometer data points were collected every 4 seconds, providing the matching time interval of the online analysis of products as a function of voltage. The measurement was started after reaching stable signals. Potential is scanned from open-circuit voltage to -1.34 V (*vs.* RHE) and then reversed back to -0.44 V (*vs.* RHE). (a.u.: arbitrary units).

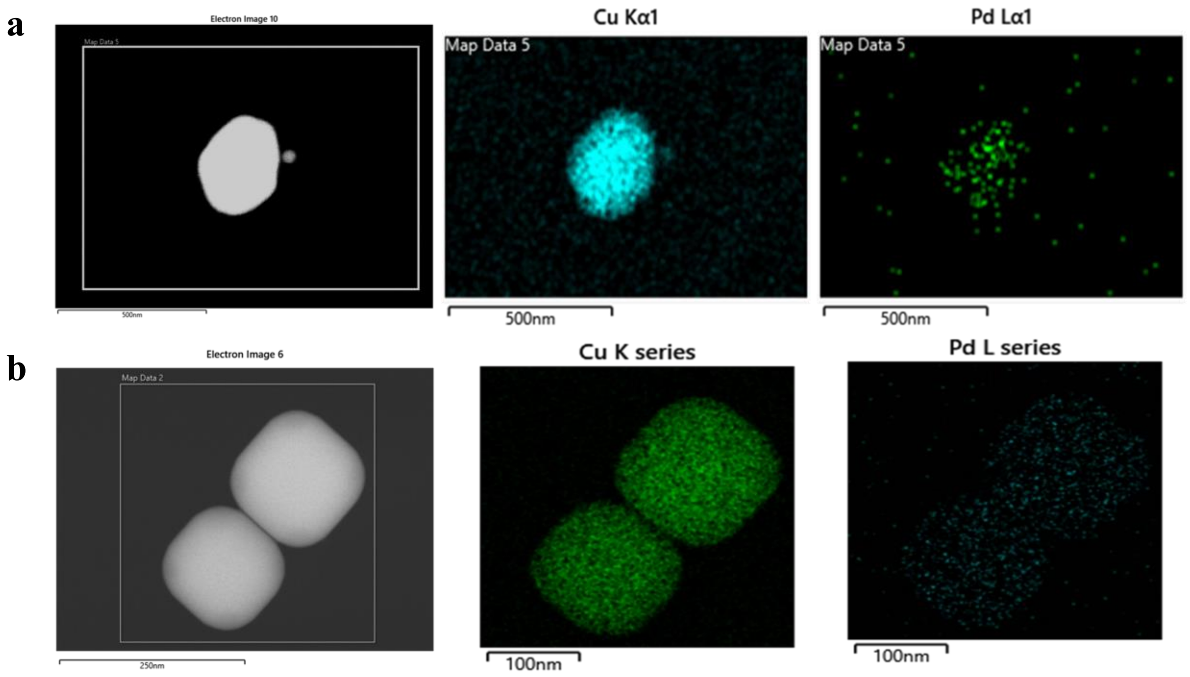

Supplementary Fig. S19: Elemental distribution in the particle. STEM-EDS mapping of the **a** Octa-Pd_1Cu SAA catalyst and **b** Cube-Pd_1Cu SAA catalyst showing the uniform distribution of Pd on the Cu shapes.

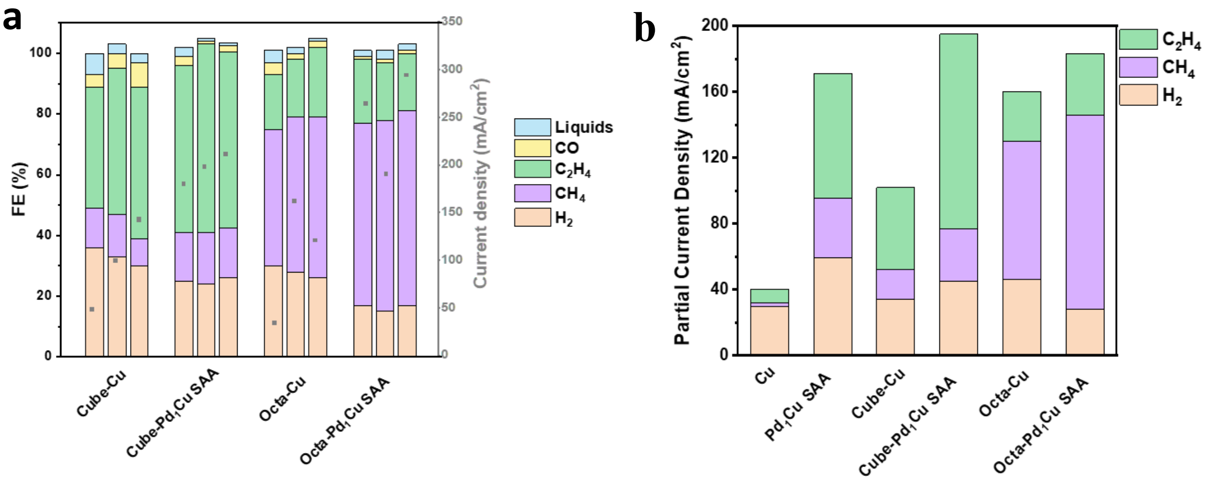

Supplementary Fig. S20: CO_2RR performance on shape-controlled catalysts. a Comparison of CO_2 reduction activity of facet-selective Cu parent catalysts (Cube and Octa) and their Pd_1Cu SAA counterparts at three different voltages- left to right (-0.9 V, -1.1 V and -1.3 V). Faradaic efficiency and current density comparison (the color codes for each product is shown at the bottom). **b** Comparison of partial current density of different products for facet selective Cu morphologies with and without their Pd single atom alloy counterpart at -1.1 V. The catalyst loading on the GDE was maintained at 220 μg/cm^2.

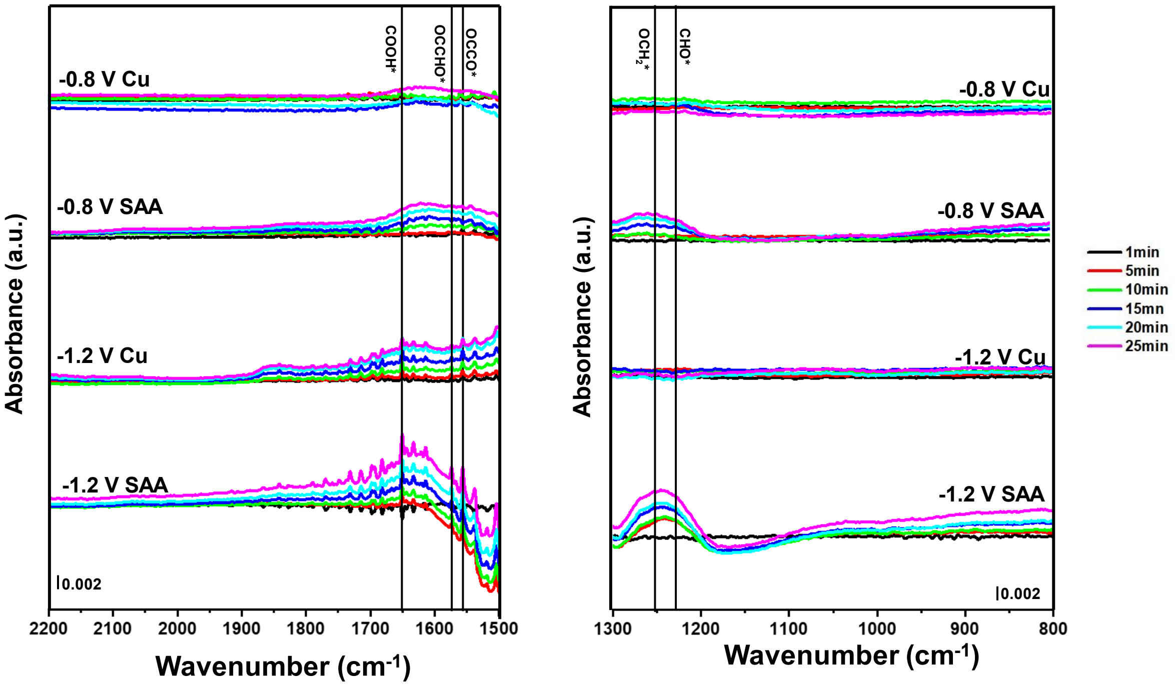

Supplementary Fig. S21: ***in-situ*** **ATR-SEIRAS absorbance spectra collected as a function of time for polycrystalline Cu and polycrystalline Pd_1Cu SAA in CO_2-saturated 0.5 M $KHCO_3$ electrolyte at -0.8 and -1.2 V.** Spectra situated between 1300 and 1500 cm^{-1} were omitted because of data distortions and overlaps caused by (bi)carbonate species. Note: For easier readability and format consistency, we put the * toward the end of the reaction intermediates in this report, and readers shall defer to the computational models to determine the actual terminal atom for those adsorbates. (a.u.: arbitrary units).

Supplementary Table S9: Summary of the IR detected intermediates during CO_2RR at different voltages over polycrystalline Cu NPs and polycrystalline Pd_1Cu SAA.

Wavenumber (cm^{-1})/ Intermediates		1650~1660 [3]	1575~1590[3]	1550~1560[3]	1245~1255[4]	1225~1235[5]
Sample /Voltage		COOH*	OCCHO*	OCCO*	OCH_2*	CHO*
SAA	-0.8 V	√	weak	weak	√	√
	-1.2 V	√	√	√	√	√
Cu	-0.8 V	weak				
	-1.2 V	√	√	√	weak	weak

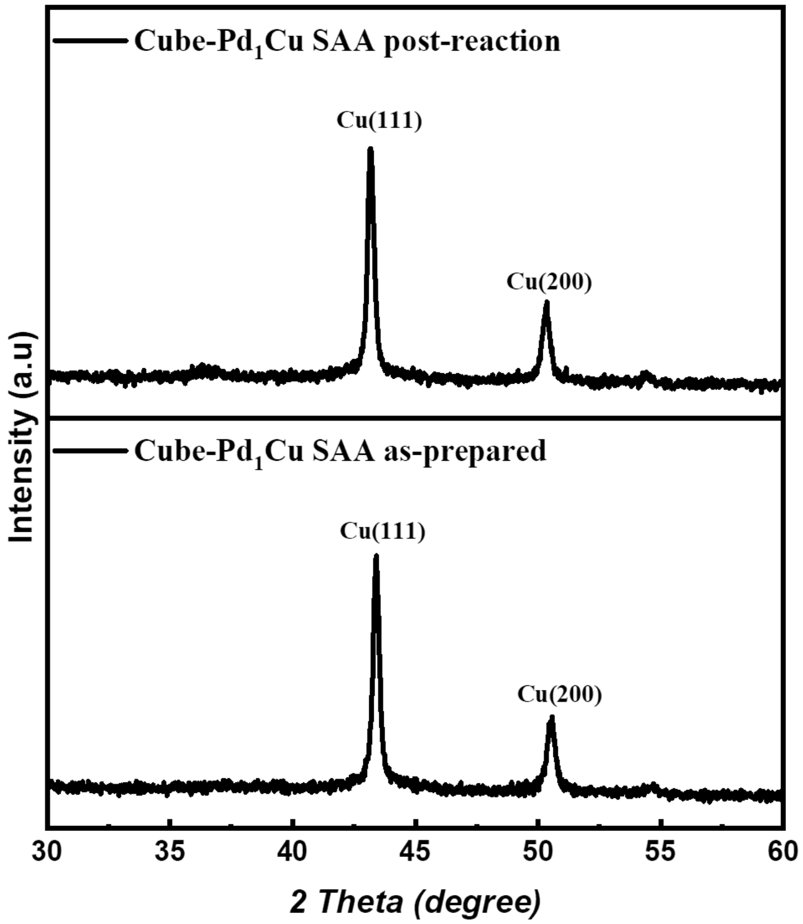

Supplementary Fig. S22a: Powder X-Ray diffraction (XRD) patterns for Cube-Pd$_1$Cu SAA as-prepared/post-reaction (0.5 M $KHCO_3$ as an electrolyte and at – 1.1 V *vs.* RHE for 30 min, a.u.: arbitrary units.)

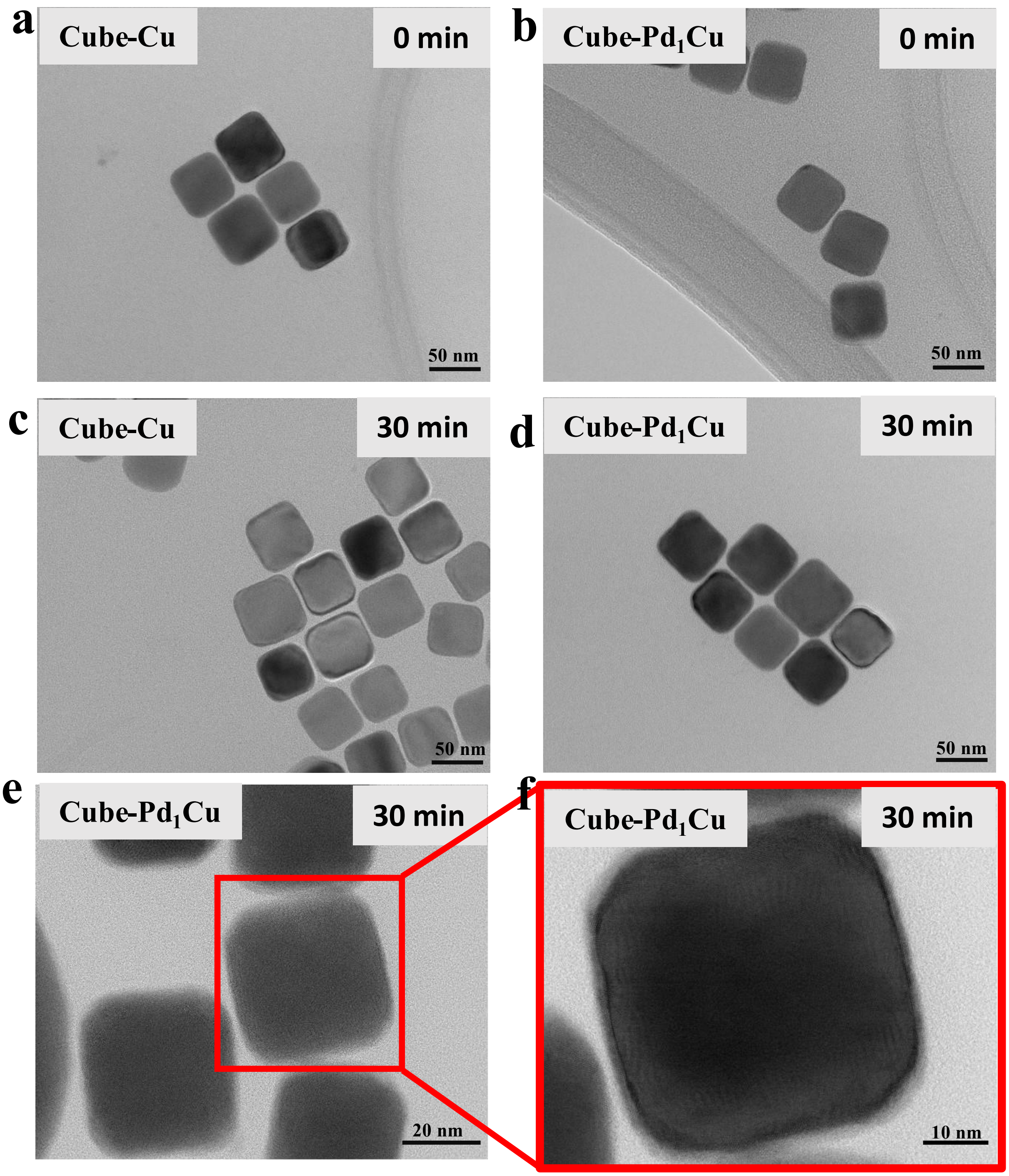

Supplementary Fig. S22b: Morphologies of the shape-controlled samples before and after CO_2RR test. a-d Representative Cube-Cu and Cube-Pd_1Cu SAA, **e-f** Representative Cube-Pd_1Cu SAA, imaged by bright-field STEM (BF-STEM) after reaction (0.5 M $KHCO_3$ as an electrolyte and at – 1.1 V *vs.* RHE for 30 min).

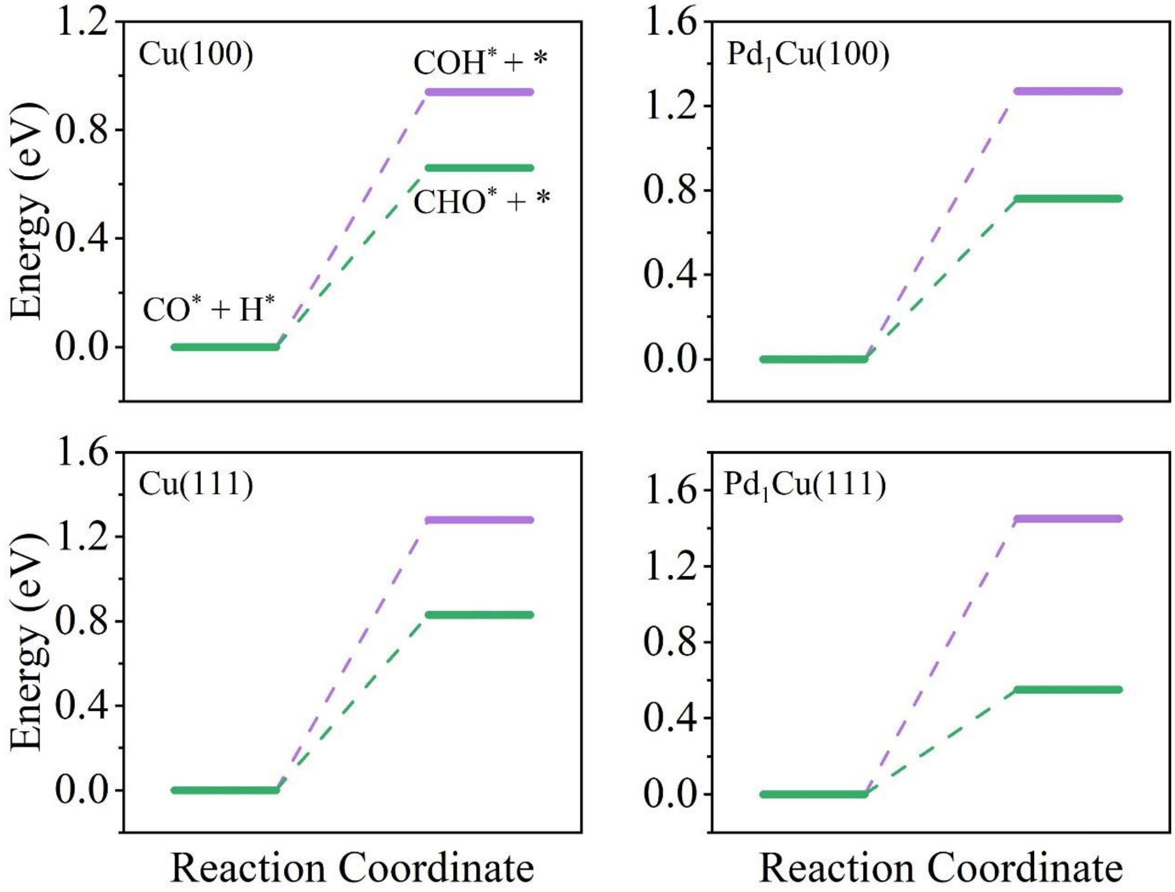

Supplementary Fig. S23: DFT energy diagram of CO^* hydrogenation to CHO^* (green line) and COH^* (purple line) over various facets. Lower energy represents a thermodynamically more favorable path.

Supplementary Table S10: Summary of the adsorption energy of COH^* over the (100) surfaces.

Surface	Adsorption site	E_{ad} (eV)
Cu(100)	**hollow**	**-3.15**
$Pd_1Cu(100)$	Cu_hollow	-3.22
	Pd_top	-2.38
	PdCu_bridge	-2.94
	**PdCu_hollow**	**-3.39**

Note: COH* at the top and bridge sites of Cu(100) are unstable and move to hollow site; COH* at the Cu_bridge and Cu_top sites of Pd1Cu(100) are unstable and move to the PdCu_hollow and PdCu_bridge sites, respectively. The most favorable adsorption site for COH* over each (100) surface is bolded.

Supplementary Table S11: Summary of the adsorption energy of COH^* over the (111) surfaces.

Surface	Adsorption site	E_{ad} (eV)
Cu(111)	**fcc**	**-2.84**
	hcp	-2.80
$Pd_1Cu(111)$	Cu_fcc	-2.65
	Cu_hcp	-2.62
	Pd_top	-2.06
	PdCu_hcp	-2.79
	**PdCu_fcc**	**-2.82**

Note: COH^* at the top and bridge sites of Cu(111) are unstable and move to the hcp and fcc sites, respectively; COH^* at the Cu_top, Cu_bridge and PdCu_bridge sites of $Pd_1Cu(111)$ are unstable and move to the PdCu_hcp site. The most favorable adsorption site for COH^* over each (111) surface is bolded.

Supplementary Table S12: Summary of the adsorption energy of CHO^* over the (100) surfaces.

Surface	Adsorption site	E_{ad} (eV)
Cu(100)	**top**	**-1.48**
	bridge	-1.47
$Pd_1Cu(100)$	Cu_top	-1.52
	Cu_bridge	-1.45
	Cu_hollow	-1.52
	**Pd_top**	**-1.97**

Note: CHO^* at the hollow site of Cu(100) is unstable and moves to the top site; CHO^* at the PdCu_bridge and PdCu_hollow sites of $Pd_1Cu(100)$ are unstable and move to the Pd_top site. The most favorable adsorption site for CHO^* over each (100) surface is bolded.

Supplementary Table S13: Summary of the adsorption energy of CHO^* over the (111) surfaces.

Surface	Adsorption site	E_{ad} (eV)
Cu(111)	**top**	**-1.36**
	bridge	-1.30
$Pd_1Cu(111)$	Cu_top	-1.25
	Cu_bridge	-1.14
	**Pd_top**	**-1.77**

Note: CHO^* at the fcc and hcp sites of Cu(111) are unstable and move to the bridge site; CHO^* at the Cu_fcc and Cu_hcp sites of $Pd_1Cu(100)$ are unstable and move to the Cu_bridge site; CHO^* at the PdCu_bridge, PdCu_fcc, and PdCu_hcp sites of $Pd_1Cu(100)$ are unstable and move to the Pd_top site. The most favorable adsorption site for CHO^* over each (111) surface is bolded.

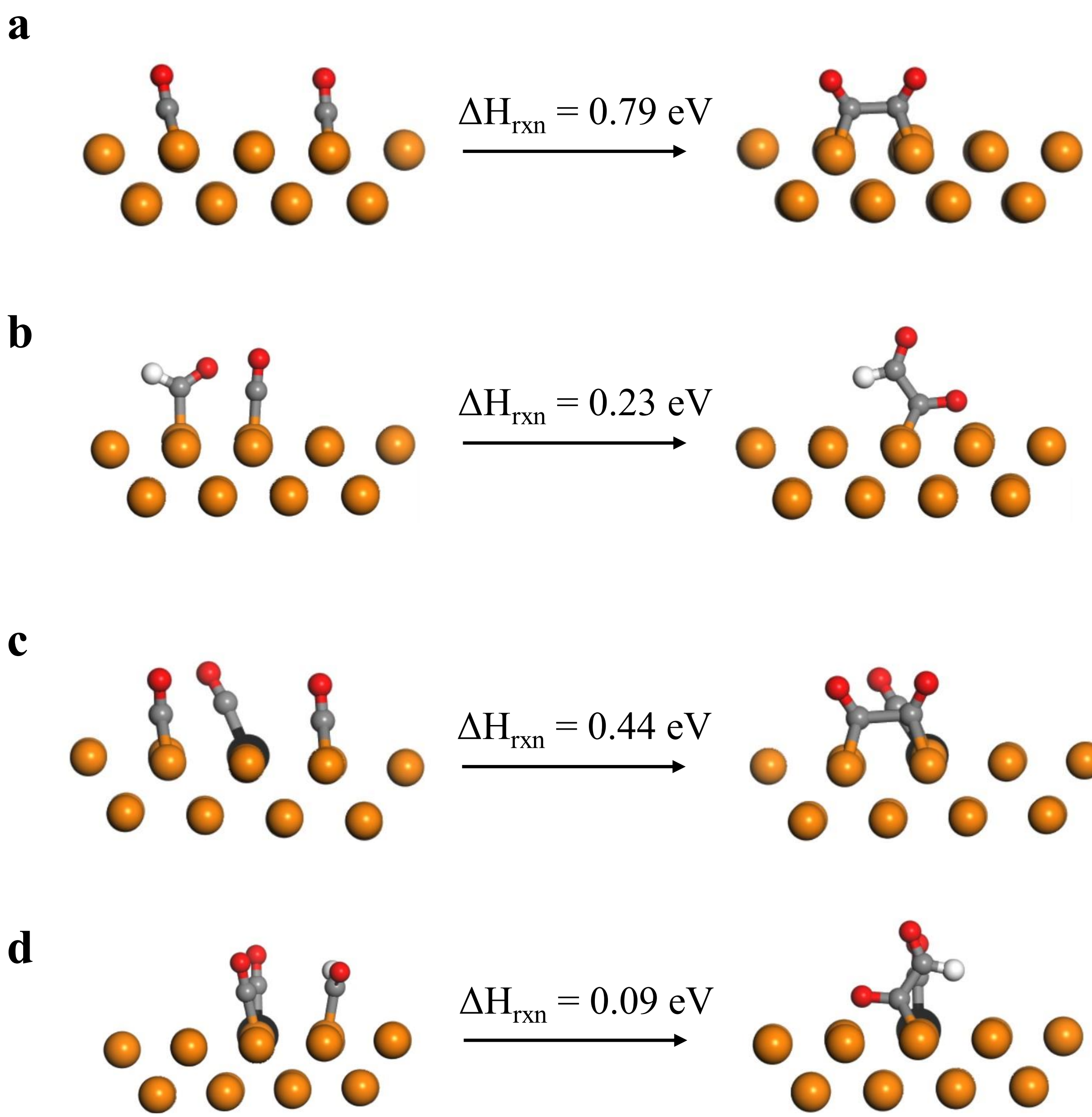

Supplementary Fig. S24: Possible C-C coupling pathways over Cu(100) and $Pd_1Cu(100)$ and the corresponding reaction energies. a $CO^* + CO^* \rightarrow OCCO^* + *$ over Cu(100). **b** $CO^* + CHO^* \rightarrow OCCHO^* + *$ over Cu(100). **c** $CO^* + CO^* \rightarrow OCCO^* + *$ over $Pd_1Cu(100)$. **d** $CO^* + CHO^* \rightarrow OCCHO^* + *$ over $Pd_1Cu(100)$. **a** and **c** incorporated two layers of explicit water solvation (i.e., 16 H_2O, not shown in the snapshot) because the hydrogen bonds from the water are necessary to stabilize $OCCO^*$ as the 'boat shape' over Cu(100) based surface[6] and generate the reasonable reaction energy of the CO dimerization. CO-CHO coupling is more likely to present in this work because (1) it has more favorable reaction energies over both Cu(100) and $Pd_1Cu(100)$ surfaces; (2) we observed the more abundant CHO^* and $OCCHO^*$ species from the in-situ ATR-SEIRAS experiment for the Cube-series catalysts that favor ethylene formation.

Supplementary Table S14: Summary of the adsorption energy of $OCCHO^*$ over the (100) surfaces.

Surface	Adsorption site	E_{ad} (eV)
Cu(100)	top	-1.38
	bridge	-1.34
	**hollow**	**-1.44**
$Pd_1Cu(100)$	Cu_bridge	-1.37
	Cu_hollow	-1.46
	**Pd_top**	**-1.80**
	PdCu_bridge	-1.62
	PdCu_hollow	-1.64

Note: The most favorable adsorption site for $OCCOH^*$ over each (100) surface is bolded.

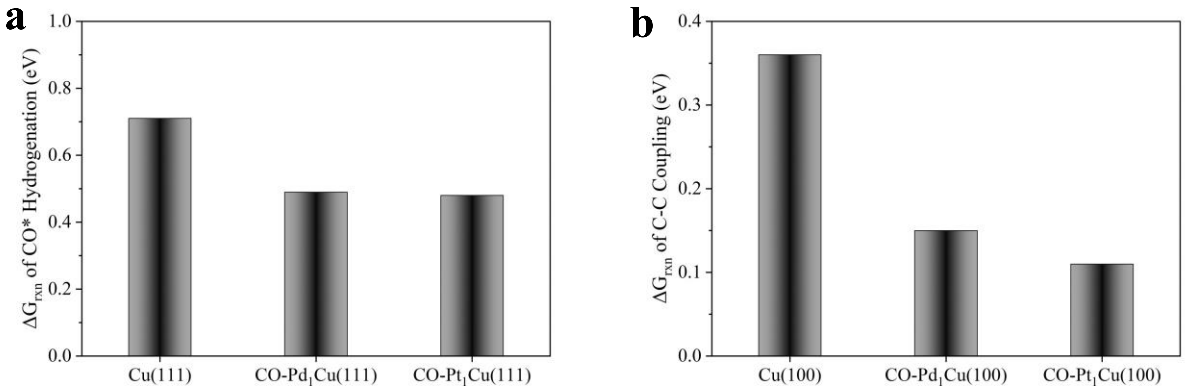

Supplementary Fig. S25: The reaction free energies of CO^* hydrogenation and C-C coupling. a The reaction free energies of CO^* hydrogenation ($H^* + CO^* \rightarrow CHO^* + *$) on Cu(111), CO-$Pd_1$Cu(111), and CO-$Pt_1$Cu(111), respectively. **b** The reaction free energies of C-C coupling ($CO^* + CHO^* \rightarrow OCCHO^* + *$) on Cu(100), CO-$Pd_1$Cu(100), and CO-$Pt_1$Cu(111), respectively.

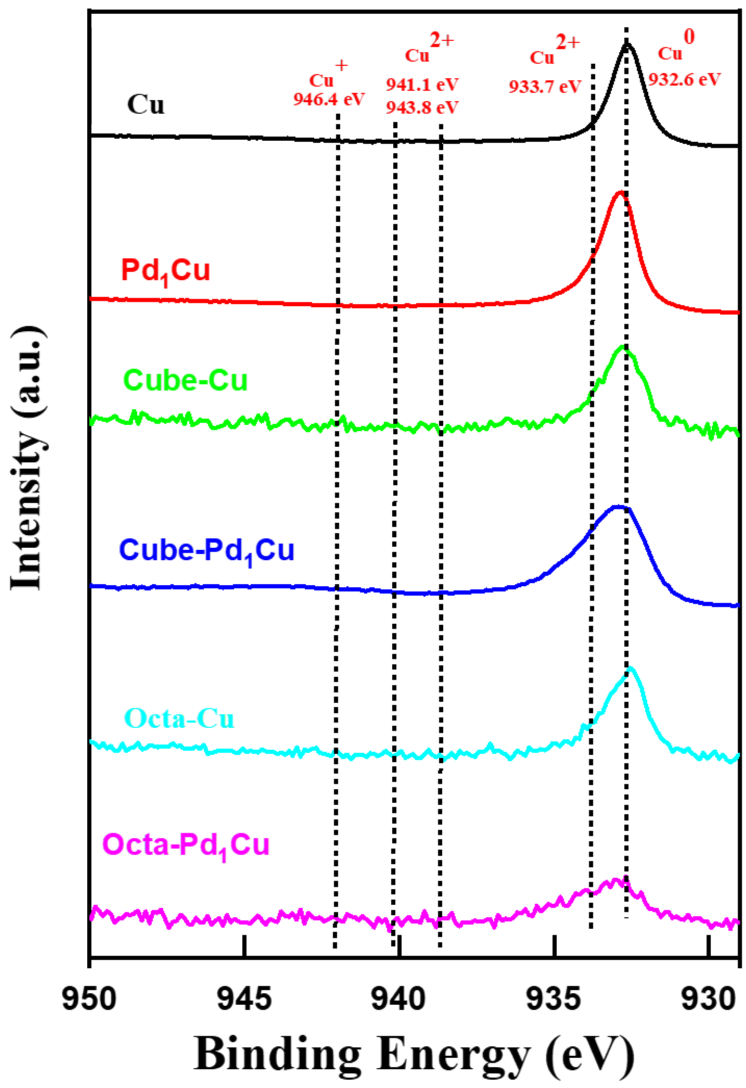

Supplementary Fig. S26: XPS spectra of Cu 2*p*3/2 peaks in Cu, Pd_1Cu SAA, Cube-Cu, Cube-Pd_1Cu SAA, Octa-Cu and Octa-Pd_1Cu SAA. (a.u.: arbitrary units).

XPS analysis of surface- Cu surface with and without Pd SAA inclusion:

We observed negligible changes in the Cu surface post-alloying with single atoms of Pd and Pt at an extremely low concentration. This is evident from the characterization section about the synthesis procedure and through XRD analysis. The proportion of Cu^{1+} and Cu^{2+} is unrecognizable through XRD and XPS studies. The high-resolution Cu 2p core level XPS spectra in Fig. S26 indicate that the samples do have insignificant amounts of Cu^{2+} (and no Cu^{1+}) species after galvanic displacement reaction, which could be attributed to the very thin adventitious oxide layer, which was so inconsequential that it remained unrecognized in XRD analysis. However, these tiny

proportions of higher valent Cu species, if any, on the surface do not contribute to product selectivity significantly, as pointed out by Buonsanti and co-workers[37] as well as Zheng *et al.*[38].

Comments on effects of other parameters on Faradaic efficiency.

Although the SAA synthesis step involves GD by ultrasonicating Cu-PGM salt mixture under the N_2 atmosphere, the possibility of partial Cu oxidation cannot be ruled out. We, however, did not see the presence of any Cu-Oxide phase in the SAA catalyst based on the XRD and XPS analysis (Fig. S24). Identifying the oxide-derived Cu is crucial as it preferentially produces ethylene (for Cu-oxide derived catalysts, selectivity ratio of C_2H_4:CH_4 >29 whereas for Cu metal catalysts C_2H_4:CH_4<2). The FE ratio between C_2H_4 and CH_4 in our catalysts suggests its metallic nature (devoid of minute Cu-oxides) in both Cu only and PGM_1Cu SAA. Consequently, our discussion pertaining to product FE improvement does not stem from changes in Cu oxidation state but solely arises because of SAA promoting effect.

Supplementary References

1. Liu, Kunlong, et al. Cu_2O-supported atomically dispersed Pd catalysts for semihydrogenation of terminal alkynes: critical role of oxide supports. *CCS Chem.* 207-214 (2019).
2. Ren, W. et al. Isolated copper–tin atomic interfaces tuning electrocatalytic CO_2 conversion. *Nat. Commun.* 12, 1449 (2021).
3. Zheng, T. *et al.* Copper-catalysed exclusive CO_2 to pure formic acid conversion via single-atom alloying. *Nat. Nanotechnol.* 16, 1386-1393 (2021).
4. Zhang, M. *et al.* Tunable Selectivity for Electrochemical CO_2 Reduction by Bimetallic Cu–Sn Catalysts: Elucidating the Roles of Cu and Sn. *ACS Catal.* 11, 11103-11108 (2021).
5. Li, M. *et al.* Mesoporous palladium–copper bimetallic electrodes for selective electrocatalytic reduction of aqueous CO_2 to CO. *J. Mater. Chem. A* 4, 4776-4782 (2016).
6. Zhu, W. *et al.* CO2 Electroreduction: Morphological and Compositional Design of Pd–Cu Bimetallic Nanocatalysts with Controllable Product Selectivity toward CO_2 Electroreduction. *Small* 14, 1870031 (2018).
7. Yin, Z. *et al.* Highly selective palladium-copper bimetallic electrocatalysts for the electrochemical reduction of CO_2 to CO. *Nano Energy* 27, 35-43 (2016).
8. Chen, D. *et al.* Tailoring the Selectivity of Bimetallic Copper–Palladium Nanoalloys for Electrocatalytic Reduction of CO_2 to CO. *ACS Appl. Energy Mater.* 1, 883-890 (2018).
9. Ma, S. *et al.* Electroreduction of Carbon Dioxide to Hydrocarbons Using Bimetallic Cu–Pd Catalysts with Different Mixing Patterns. *J. Am. Chem. Soc.* 139, 47-50 (2017).
10. Takashima, T., Suzuki, T. & Irie, H. Electrochemical carbon dioxide reduction on copper-modified palladium nanoparticles synthesized by underpotential deposition. *Electrochim. Acta* 229, 415-421 (2017).
11. Rahaman, M. *et al.* Selective n-propanol formation from CO_2 over degradation-resistant activated PdCu alloy foam electrocatalysts. *Green Chem.* 22, 6497-6509 (2020).

12. Mun, Y. *et al.* Cu-Pd alloy nanoparticles as highly selective catalysts for efficient electrochemical reduction of CO_2 to CO. *Appl. Catal. B: Environ.* 246, 82-88 (2019).
13. Chen, D. *et al.* Surface composition dominates the electrocatalytic reduction of CO_2 on ultrafine CuPd nanoalloys. *Carbon Energy* 2, 443-451 (2020).
14. Weng, Z. *et al.* Self-Cleaning Catalyst Electrodes for Stabilized CO_2 Reduction to Hydrocarbons. *Angew. Chem. Int. Ed.* 56, 13135-13139 (2017).
15. Zhao, X., Luo, B., Long, R., Wang, C. & Xiong, Y. Composition-dependent activity of Cu–Pt alloy nanocubes for electrocatalytic CO_2 reduction. *J. Mater. Chem. A* 3, 4134-4138 (2015).
16. Lyu, Z. *et al.* Kinetically Controlled Synthesis of Pd–Cu Janus Nanocrystals with Enriched Surface Structures and Enhanced Catalytic Activities toward CO_2 Reduction. *J. Am. Chem. Soc.* 143, 149-162 (2021).
17. Guo, X. *et al.* Composition dependent activity of Cu–Pt nanocrystals for electrochemical reduction of CO_2. *Chem. Commun.* 51, 1345-1348 (2015).
18. Jouny, Matthew, et al. High-rate electroreduction of carbon monoxide to multi-carbon products. *Nat. Catal.* 1, 748-755 (2018).
19. Burdyny, Thomas, et al. Nanomorphology-enhanced gas-evolution intensifies CO_2 reduction electrochemistry. *ACS Sustain. Chem. Eng.* 5, 4031-4040(2017).
20. Dinh, Cao-Thang, et al. CO_2 electroreduction to ethylene via hydroxide-mediated copper catalysis at an abrupt interface. *Science* 360, 783-787 (2018).
21. Li, Jun, et al. Enhanced multi-carbon alcohol electroproduction from CO via modulated hydrogen adsorption. *Nat. Commun.* 11, 3685, (2020).
22. Lu, Xiaofei, et al. Product Distribution Control Guided by a Microkinetic Analysis for CO Reduction at High-Flux Electrocatalysis Using Gas-Diffusion Cu Electrodes. *ACS Catal.* 13, 1791-1803 (2023).
23. Li, Jun, et al. Constraining CO coverage on copper promotes high-efficiency ethylene electroproduction. *Nat. Catal.* 2, 1124-1131. 4 (2019).
24. Trasatti, S., and O. A. Petrii. Real surface area measurements in electrochemistry. J. Electroanal. Chem. 327, 353-376 (1992).
25. Clark, Ezra L., et al. Data acquisition protocols and reporting standards for studies of the electrochemical reduction of carbon dioxide. *ACS Catal.* 8, 6560-6570 (2018).
26. Kresse, G. & Hafner, J. Ab initio molecular dynamics for liquid metals. *Phys. Rev. B* 47, 558-561 (1993).
27. Kresse, G. & Furthmüller, J. Efficiency of ab-initio total energy calculations for metals and semiconductors using a plane-wave basis set. *Comput. Mater. Sci.* 6, 15-50 (1996).
28. Hammer, B., Hansen, L. B. & Nørskov, J. K. Improved adsorption energetics within density-functional theory using revised Perdew-Burke-Ernzerhof functionals. *Phys. Rev. B* 59, 7413-7421 (1999).
29. Monkhorst, H. J. & Pack, J. D. Special points for Brillouin-zone integrations. *Phys. Rev. B* 13, 5188-5192 (1976).
30. Gajdoš, M. & Hafner, J. CO adsorption on Cu(111) and Cu(001) surfaces: Improving site preference in DFT calculations. *Surf. Sci.* 590, 117-126 (2005).
31. Behler, J. Perspective: Machine learning potentials for atomistic simulations. *J. Chem. Phys.* 145, 170901 (2016).

32. Grimme, S., Antony, J., Ehrlich, S. & Krieg, H. A consistent and accurate ab initio parametrization of density functional dispersion correction (DFT-D) for the 94 elements H-Pu. *J. Chem. Phys.* 132, 154104 (2010).
33. D.R. Stull and H. Prophet. *JANAF thermochemical tables [electronic resource] / D.R. Stull and H. Prophet, project directors*. (U.S. Dept. of Commerce, National Bureau of Standards, 1971).
34. Nørskov, J. K. *et al.* Trends in the Exchange Current for Hydrogen Evolution. *J. Electrochem. Soc.* 152, J23 (2005).
35. Tang, J.-X. *et al.* Screw-like PdPt nanowires as highly efficient electrocatalysts for methanol and ethylene glycol oxidation. *J. Mater. Chem. A* 6, 2327-2336 (2018).
36. Firet, N. J. & Smith, W. A. Probing the Reaction Mechanism of CO_2 Electroreduction over Ag Films via Operando Infrared Spectroscopy. *ACS Catal.* 7, 606-612 (2017).
37. Huang, J. *et al.* Potential-induced nanoclustering of metallic catalysts during electrochemical CO_2 reduction. *Nat. Commun.* 9, 3117 (2018).
38. Zheng, T. *et al.* Copper-catalysed exclusive CO_2 to pure formic acid conversion via single-atom alloying. *Nat. Nanotechnol.* 16, 1386-1393 (2021).
